# Establishment and Validation of a Prognostic Nomogram for Predicting Postoperative Overall Survival in Advanced Stage III–IV Colorectal Cancer Patients

**DOI:** 10.1002/cam4.70385

**Published:** 2024-11-15

**Authors:** Pengwei Lou, Dongmei Luo, Yuting Huang, Chen Chen, Shuai Yuan, Kai Wang

**Affiliations:** ^1^ Department of Big Data, College of Information Engineering Xinjiang Institute of Engineering Urumqi Xinjiang Uygur Autonomous Region People's Republic of China; ^2^ Department of Medical Administration Cancer Hospital Affiliated With Xinjiang Medical University Urumqi Xinjiang Uygur Autonomous Region People's Republic of China; ^3^ Department of Medical Administration Traditional Chinese Medicine Hospital Affiliated With Xinjiang Medical University Urumqi Xinjiang Uygur Autonomous Region People's Republic of China; ^4^ College of Public Health Xinjiang Medical University Urumqi Xinjiang Uygur Autonomous Region People's Republic of China; ^5^ Department of Urology Cancer Hospital Affiliated With Xinjiang Medical University Urumqi Xinjiang Uygur Autonomous Region People's Republic of China

**Keywords:** colorectal cancer, nomogram, overall survival, prognosis

## Abstract

**Background:**

Most colorectal cancer (CRC) patients are at an advanced stage when they are first diagnosed. Risk factors for predicting overall survival (OS) in advanced stage CRC patients are crucial, and constructing a prognostic nomogram model is a scientific method for survival analysis.

**Methods:**

A total of 2956 advanced stage CRC patients were randomised into training and validation groups at a 7:3 ratio. Univariate and multivariate Cox proportional hazards regression analyses were used to screen risk factors for OS and subsequently construct a prognostic nomogram model for predicting 1‐, 3‐, 5‐, 8‐ and 10‐year OS of advanced stage CRC patients. The performance of the model was demonstrated by the area under the curve (AUC) values, calibration curves and decision curve analysis (DCA). Kaplan–Meier curves were used to plot the survival probabilities for different strata of each risk factor.

**Results:**

There was no statistically significant difference (*p* > 0.05) in the 32 clinical variables between patients in the training and validation groups. Univariate and multivariate Cox proportional hazards regression analyses demonstrated that age, location, TNM, chemotherapy, liver metastasis, lung metastasis, MSH6, CEA, CA199, CA125 and CA724 were risk factors for OS. We estimated the AUC values for the nomogram model to predict 1‐, 3‐, 5‐, 8‐ and 10‐year OS, which in the training group were 0.826 (95% CI: 0.807–0.845), 0.836 (0.819–0.853), 0.839 (0.820–0.859), 0.835 (0.809–0.862) and 0.825 (0.779–0.870) respectively; in the validation group, the corresponding AUC values were 0.819 (0.786–0.852), 0.831 (0.804–0.858), 0.830 (0.799–0.861), 0.815 (0.774–0.857) and 0.802 (0.723–0.882) respectively. Finally, the 1‐, 3‐, 5‐, 8‐ and 10‐year OS rates for advanced stage CRC patients were 73.4 (71.8–75.0), 49.5 (47.8–51.4), 43.3 (41.5–45.2), 40.1 (38.1–41.9) and 38.6 (36.6–40.8) respectively.

**Conclusion:**

We constructed and validated an original nomogram for predicting the postoperative OS of advanced stage CRC patients, which can help facilitates physicians to accurately assess the individual survival of postoperative patients and identify high‐risk patients.

## 1 Introduction

Colorectal cancer (CRC) is one of the most common digestive tract tumours worldwide [1]. In 2020, the number of new CRC cases reached 1.932 million globally, which is the third highest among all cancers and accounts for 10.0% of new cancer cases. During the same period, China ranked second, with 555,000 new cases of CRC, accounting for approximately 12.2% of the total number of new cancer cases and 28.8% of the new CRC cases worldwide. There were 286,000 deaths from CRC, ranking fifth among cancers, accounting for approximately 9.5% of cancer deaths and 30.6% of CRC deaths worldwide [2]. Therefore, the importance of research on CRC treatment and prognosis in terms of patient survival cannot be overemphasised. Although the clinical outcomes of CRC patients have improved significantly, the prognosis is still unsatisfactory and the mortality rate of CRC remains high, especially in patients with distant metastases [3, 4].

Adenocarcinoma originating from the epithelial cells of the colorectal mucosa is the most common type of colorectal tumour, and it accounts for more than 90% of all CRC cases [5, 6, 7, 8, 9]. Early‐stage CRC is mostly asymptomatic, and approximately 60% to 80% of patients are diagnosed with CRC at an advanced stage, which have a poorer prognosis and higher mortality rate than those diagnosed at an early stage [10, 11, 12, 13, 14, 15, 16, 17]. Therefore, screening risk factors for overall survival (OS) in advanced stage CRC patients and exploring effective treatments are critical for prolonging survival.

Over the past decade, nomograms have gained widespread acceptance as a unique and reliable method for the individualised prediction of tumour prognosis [18]. On the basis of a multivariate linear regression model, the nomogram integrates multiple clinical predictors and shows the quantitative relationships among these individual predictors to accurately predict OS for individual patients, helping clinicians optimise individualised treatment regimens and assess treatment outcomes [19]. Many such studies on CRC have been reported recently, but they have several limitations: (1) Most of the studies are based on the Surveillance, Epidemiology, and End Results (SEER) public database established by the U.S. National Cancer Institute, and these data resources are not representative of CRC prognosis in other regions [20, 21, 22]; (2) the sample sizes of the local studies are relatively small and the influencing factors are not very comprehensive [23, 24, 25]; (3) several studies have performed nomogram prognostic modelling for all CRC patients (stages I–IV) or for early‐stage (stages I and II) patients, whereas fewer studies have focused on advanced stage (stages III and IV) patients [19, 26, 27, 28]. In this study, we analysed the postoperative OS of advanced stage CRC patients in Chinese hospitals and explored the risk factors affecting prognosis by constructing a nomogram model.

## 2 Patients and Methods

### 2.1 Data Sources and Selection Criteria

The source of data and information for this study was CRC patients who were diagnosed between 1 January 2010 and 31 December 2019 at the Cancer Hospital Affiliated with Xinjiang Medical University.

CRC patients were divided according to the American Joint Committee of Cancer (AJCC) TNM system eighth edition criteria into early‐stage (stages I and II) and advanced stage (stages III and IV) CRC at diagnosis [14, 29].

**FIGURE 1:**
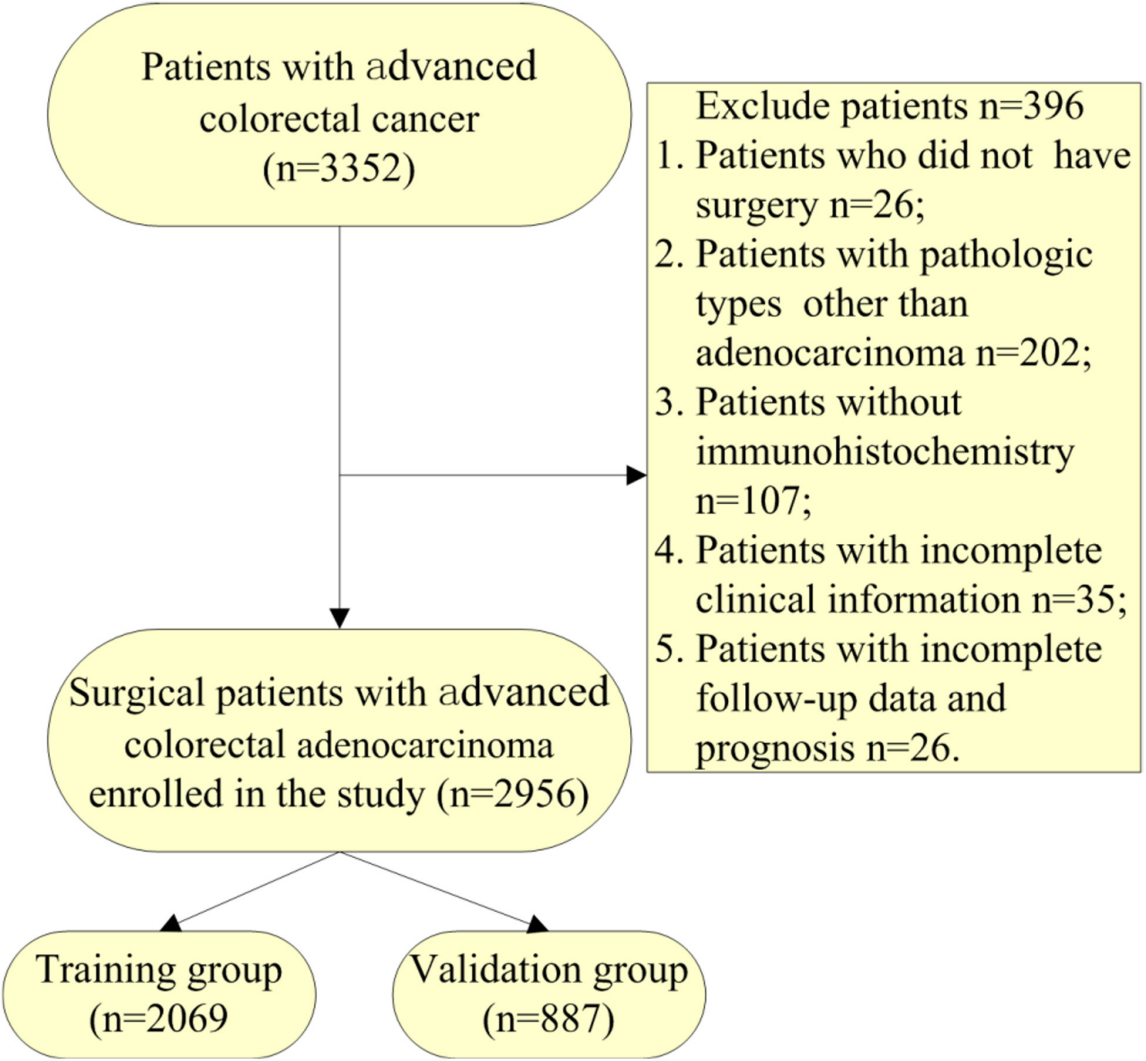
Screening flowchart for advanced stage colorectal cancer (CRC) patients.

The screening criteria for advanced stage CRC patients were developed to obtain representative research subjects. The inclusion criteria were as follows: (1) primary site of malignancy in the colorectum and absence of primary cancer elsewhere; (2) definitive pathological diagnosis and (3) TNM stages III and IV. The exclusion criteria were as follows: (1) no surgical intervention or more than one surgical treatment; (2) pathological type other than adenocarcinoma; (3) incomplete basic patient information and clinical test results and (4) patients with incomplete follow‐up data whose prognosis was unknown. The screening process for the advanced stage CRC patients is shown in Figure 1. There were 3352 patients with stage III–IV CRC diagnosed by pathologic testing. We subsequently excluded 26 patients who did not undergo surgical treatment; 202 non‐adenocarcinoma patients with pathologic types of squamous carcinoma, adenosquamous carcinoma, small‐cell carcinoma and undifferentiated carcinoma; 107 patients who did not undergo immunohistochemistry testing for genetic proteins; 35 patients whose clinical testing data were lacking and 26 patients whose follow‐up data and prognosis were not available. To ensure the authenticity and scientific validity of the study, we excluded 396 patients who did not meet the criteria, and a total of 2956 advanced stage CRC patients were ultimately included in this study. These patients were randomly assigned to the training group (*n* = 2069) and the internal validation group (*n* = 887) at a ratio of 7:3.

The start of follow‐up was the time of the patient's first surgery confirmed by pathology, the follow‐up deadline for the study participants was 31 December 2022, the follow‐up duration was 1–156 months and the median follow‐up duration was 34 months. This study was approved by the Ethics Committee of the Cancer Hospital Affiliated with Xinjiang Medical University (Approval Number: K‐2023005), all patients provided written informed consent, all procedures performed in studies involving human participants were conducted in accordance with the Helsinki Declaration.

### 2.2 Research Variables

This study combines patients' clinical information and various test indicators. A total of 32 study variables, which were categorised into the following five main groups, were included:

1. Basic patient information, including age and gender.
2. Clinical information about the CRC: location, diameters, TNM, T, N, M, tumour differentiation, liver metastasis, lung metastasis, bone metastasis, splenic metastasis, metastasis to other body parts, nerve invasion, vascular invasion, extramural vascular invasion and lymph node metastasis.
3. Treatment modalities for CRC patients: radiotherapy, chemotherapy, surgical treatment and circumferential resection margins. After surgery, treatment can be combined with radiotherapy or chemotherapy according to the actual condition of the patient.
4. The proteins of the genes mutL homologue 1 (MLH1), mutS homologue 2 (MSH2), mutS homologue 6 (MSH6) and post‐meiotic segregation increased 2 (PMS2) were immunohistochemically examined and the stability of microsatellites was determined on the basis of the results. The microsatellites were categorised into three classes, with positivity indicating gene expression and negativity indicating deletion. When all four genes were positive, microsatellite stability (MSS) was defined; when only one gene was negative, it was classified as microsatellite instability–low (MSI‐L) and when ≥ 2 genes were negative, microsatellite instability–high (MSI‐H) was determined.
5. Serum tumour biomarkers: carcinoembryonic antigen (CEA), cancer antigen 199 (CA199), cancer antigen 125 (CA125), alpha‐fetoprotein (AFP) and cancer antigen 724 (CA724). The corresponding reference standards are shown in Table 1. The value was set to ‘normal’ if the test value is within the normal reference value range; otherwise, it was set to ‘high’.

**TABLE 1:** Serum tumour biomarker reference standards.

Variables	Reference standard			
	Normal value reference standard		Outlier reference standard	
CEA (ng/mL)	< 5	Normal	≥ 5	High
CA199 (U/mL)	< 37	Normal	≥ 37	High
CA125 (KU/L)	< 35	Normal	≥ 35	High
AFP (μg/L)	< 25	Normal	≥ 25	High
CA724 (U/mL)	< 6	Normal	≥ 6	High

### 2.3 Statistical Analysis

When comparing the baseline characteristics between the training group and the validation group, the chi‐squared test or Fisher's exact test was used for categorical variables. We subsequently constructed a nomogram risk prediction model. Univariate Cox proportional hazards regression analysis was implemented to evaluate the effects of some essential factors on advanced stage CRC patients. Variables with *p* < 0.05 in the univariate analysis were included in the multivariate Cox proportional hazards regression analysis and hazard ratios (HRs) were calculated to measure the impact of each independent prognostic factor. The identified risk factors were included in the nomogram model for predicting 1‐, 3‐, 5‐, 8‐ and 10‐year OS of patients with advanced stage CRC.

We used three methods to assess the proposed nomogram. First, a receiver operating characteristic (ROC) curve was used to evaluate the discrimination ability of the nomogram model. The area under the curve (AUC) ranges from 0.5 to 1, with values closer to 1 indicating better performance of the nomogram model. Second, a calibration curve was used to evaluate the agreement between the model predictions and the actual values. A greater overlap between the calibration curve and the diagonal straight line indicates a smaller error between the model‐predicted and actual values. Finally, decision curve analysis (DCA) was performed in this study as a method for assessing the ability of the predictive model to visualise clinical outcomes and was conducted to compare the net benefit of the predictive and prognostic nomograms. Kaplan–Meier curves were used to show the fluctuating trend of survival probabilities for each risk factor in different stratifications.

Open source R software version 4.3.1 (http://cran.r‐project.org/) was used for data processing, statistical analysis and modelling. In this study, the following R packages were downloaded to construct a nomogram, plot ROC curves, perform calibration and DCA and draw Kaplan–Meier curves: ‘Hmisc’, ‘survival’, ‘rms’, ‘pROC’, ‘survivalROC’, ‘MASS’ and ‘rmda’. All of the statistical tests were two‐sided, with *p*s < 0.05 considered statistically significant.

## 3 Results

### 3.1 Clinical Information for the Training and Validation Groups

The 2956 advanced stage CRC patients were randomly divided into 2069 patients in the training group and 887 patients in the validation group. Compared with the baseline data (a total of 32 influencing factors were selected) of patients in the training and validation groups, the differences were not statistically significant (*p* > 0.05), except for the ‘radiotherapy’ variable, which indicated that the allocation of patients into the training and validation groups was scientifically reasonable and comparable and could be analysed and studied further. The specific results are shown in Table 2.

**TABLE 2:** Comparison of baseline data for advanced stage CRC patients in the training and validation groups.

Variables	All patients *N* = 2956 (%)	Training group *N* = 2069 (%)	Validation group *N* = 887 (%)	^*χ2*^	*p*
Age					
< 60	1399 (47.33)	987 (47.70)	412 (46.45)	0.393	0.531
≥ 60	1557 (52.67)	1082 (52.30)	475 (53.55)		
Gender					
Male	1695 (57.34)	1180 (57.03)	515 (58.06)	0.268	0.604
Female	1261 (42.66)	889 (42.97)	372 (41.94)		
Location					
Colon	1356 (45.87)	957 (46.25)	399 (44.98)	0.404	0.525
Rectum	1600 (54.13)	1112 (53.75)	488 (55.02)		
TNM					
III	1499 (50.71)	1029 (49.73)	470 (52.99)	2.629	0.105
IV	1457 (49.29)	1040 (50.27)	417 (47.01)		
T					
T2	138 (4.67)	101 (4.88)	37 (4.17)	1.023	0.600
T3	1113 (37.65)	784 (37.89)	329 (37.09)		
T4	1705 (57.68)	1184 (57.23)	521 (58.74)		
N					
N0	250 (8.46)	178 (8.60)	72 (8.12)	1.524	0.467
N1–2	2441 (82.58)	1714 (82.84)	727 (81.96)		
N3–4	265 (8.96)	177 (8.56)	8 (0.902)		
M					
M0	949 (32.10)	647 (31.27)	302 (34.05)	2.195	0.138
M1	2007 (67.90)	1422 (68.73)	585 (65.95)		
Radiotherapy					
No	1649 (55.78)	1127 (54.47)	522 (58.85)	4.827	0.028
Yes	1307 (44.22)	942 (45.53)	365 (41.15)		
Chemotherapy					
No	268 (9.07)	201 (9.71)	67 (7.55)	3.518	0.061
Yes	2688 (90.93)	1868 (90.29)	820 (92.45)		
Liver metastasis					
No	1915 (64.78)	1319 (63.75)	596 (67.19)	3.224	0.073
Yes	1041 (35.22)	750 (36.25)	291 (32.81)		
Lung metastasis					
No	2281 (77.17)	1577 (76.22)	704 (79.37)	3.492	0.062
Yes	675 (22.83)	492 (23.78)	183 (20.63)		
Bone metastasis					
No	2488 (84.17)	1725 (83.37)	763 (86.02)	3.264	0.071
Yes	468 (15.83)	344 (16.63)	124 (13.98)		
Splenic metastasis					
No	2944 (99.59)	2062 (99.66)	882 (99.44)	0.780	0.377
Yes	12 (0.41)	7 (0.34)	5 (0.56)		
Metastasis to other body parts					
No	1910 (64.61)	1334 (64.48)	576 (64.94)	0.058	0.810
Yes	1046 (35.39)	735 (35.52)	311 (35.06)		
Diameters					
< 5.5	1654 (55.95)	1151 (55.63)	503 (56.71)	0.292	0.589
≥ 5.5	1302 (44.05)	918 (44.37)	384 (43.29)		
Tumour differentiation					
High	235 (7.95)	177 (8.56)	58 (6.54)	3.461	0.177
Middle	2268 (76.73)	1576 (76.17)	692 (33.45)		
Low	453 (15.32)	316 (15.27)	137 (6.62)		
Nerve invasion					
No	2119 (71.68)	1483 (71.68)	636 (71.70)	< 0.001	0.989
Yes	837 (28.32)	586 (28.32)	251 (28.30)		
Vascular invasion					
No	2089 (70.67)	1462 (70.66)	627 (70.69)	< 0.001	0.989
Yes	867 (29.33)	607 (29.34)	260 (29.31)		
Extramural vascular invasion					
No	2831 (95.77)	1976 (95.51)	855 (96.39)	1.207	0.272
Yes	125 (4.23)	93 (4.49)	32 (3.61)		
Lymph node metastasis					
No	208 (7.04)	150 (7.25)	58 (6.54)	0.480	0.489
Yes	2748 (92.96)	1919 (92.75)	829 (93.46)		
Surgical method					
Palliative resection	214 (7.24)	149 (7.20)	65 (7.33)	0.172	0.918
Radical resection	2605 (88.13)	1822 (88.06)	783 (88.27)		
Enlarging resection	137 (4.63)	98 (4.74)	39 (4.40)		
Circumferential resection margin					
No	2770 (93.71)	1939 (93.72)	831 (93.69)	0.001	0.975
Yes	186 (6.29)	130 (6.28)	56 (6.31)		
MLH1					
Negative	121 (4.09)	86 (4.16)	35 (3.95)	0.070	0.791
Positive	2835 (95.91)	1983 (95.84)	852 (96.05)		
MSH2					
Negative	70 (2.37)	53 (2.56)	17 (1.92)	1.117	0.290
Positive	2886 (97.63)	2016 (97.44)	870 (98.08)		
MSH6					
Negative	71 (2.40)	50 (2.42)	21 (2.37)	0.006	0.936
Positive	2885 (97.60)	2019 (97.58)	866 (97.63)		
PMS2					
Negative	89 (3.01)	63 (3.04)	26 (2.93)	0.027	0.868
Positive	2867 (96.99)	2006 (96.96)	861 (97.07)		
Microsatellite					
Stability	2725 (92.19)	1901 (91.88)	824 (92.90)	1.029	0.598
Low instability	133 (4.50)	98 (4.74)	35 (3.95)		
Highly unstable	98 (3.31)	70 (3.38)	28 (3.16)		
CEA					
Normal	1296 (43.84)	895 (43.26)	401 (45.21)	0.960	0.327
High	1660 (56.16)	1174 (56.74)	486 (54.79)		
CA199					
Normal	2021 (68.37)	1399 (67.62)	622 (70.12)	1.804	0.179
High	935 (31.63)	670 (32.38)	265 (29.88)		
CA125					
Normal	2383 (80.62)	1664 (80.43)	719 (81.06)	0.160	0.689
High	573 (19.38)	405 (19.57)	168 (18.94)		
AFP					
Normal	2937 (99.36)	2057 (99.42)	880 (99.21)	0.425	0.514
High	19 (0.64)	12 (0.58)	7 (0.79)		
CA724					
Normal	1946 (65.83)	1359 (65.68)	587 (66.18)	0.067	0.795
High	1010 (34.17)	710 (34.32)	300 (33.82)		

*Note:* Data are presented as *n* (%).

Abbreviations: AFP, alpha‐fetoprotein; CA125, cancer antigen 125; CA199, cancer antigen 199; CA724, cancer antigen 724CEA, carcinoembryonic antigen; MLH1, mutL homologue 1; MSH2, mutS homologue 2; MSH6, mutS homologue 6; PMS2, post‐meiotic segregation increased 2.

### 3.2 Univariate and Multivariate Risk Factor Screening

**FIGURE 2:**
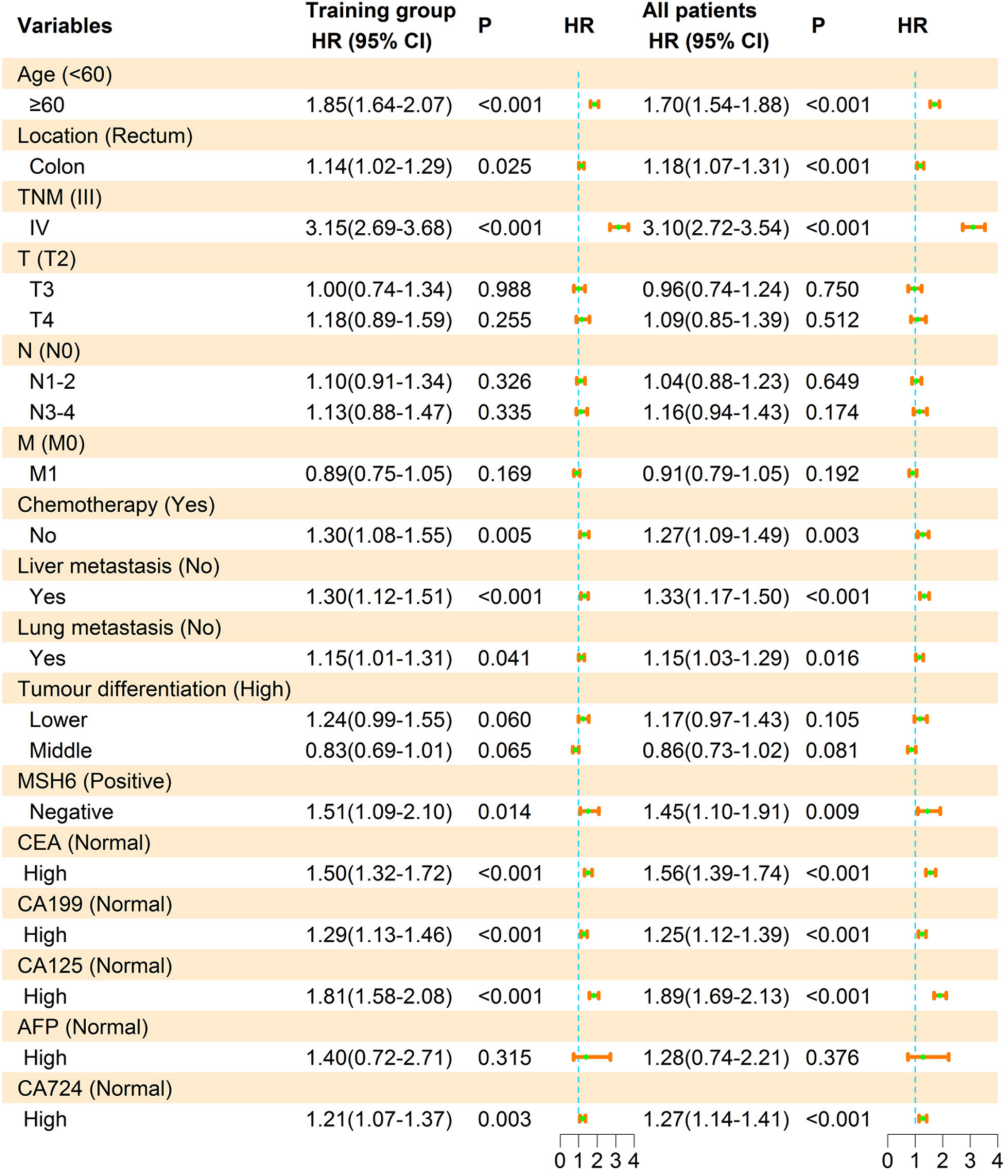
Multivariate Cox proportional hazards regression analysis and corresponding forest plots for overall survival (OS) in different groups of advanced stage colorectal cancer (CRC) patients.

The 16 statistically significant risk factors (including age, location, TNM, T, N, M, chemotherapy, liver metastasis, lung metastasis, tumour differentiation, MSH6, CEA, CA199, CA125, AFP and CA724) screened by the univariate Cox proportional hazards regression analysis for the training group were included in the multivariate Cox proportional hazards regression analysis, the results are presented in Table S1 and Figure 2. The final risk factors for OS in both the training group and all patients with advanced stage CRC were identified as age (≥ 60), location (colon), TNM (IV), chemotherapy (no), liver metastasis (yes), lung metastasis (yes), MSH6 (negative), CEA (high), CA199 (high), CA125 (high), CA724 (high).

In the training group, the rankings based on HR were as follows: TNM [IV, HR = 3.15 (95% CI: 2.69–3.68)], age [≥ 60, 1.85 (1.64–2.07)], CA125 [high, 1.81 (1.58–2.08)], MSH6 [negative, 1.51 (1.09–2.10)], CEA [high, 1.50 (1.32–1.72)], chemotherapy [no, 1.30 (1.08–1.55)], liver metastasis [yes, 1.30 (1.12–1.51)], CA199 [high, 1.29 (1.13–1.46)], CA724 [high, 1.21 (1.07–1.37)], lung metastasis [yes, 1.15 (1.01–1.31)], location [colon, 1.14 (1.02–1.29)].

In all patients, the descending order was TNM [IV, HR = 3.10 (95% CI: 2.72–3.54)], CA125 [high, 1.89 (1.69–2.13)], age [≥ 60, 1.70 (1.54–1.88)], CEA [high, 1.56 (1.39–1.74)], MSH6 [negative, 1.45 (1.10–1.91)], liver metastasis [yes, 1.33 (1.17–1.50)], chemotherapy [no, 1.27 (1.09–1.49)], CA724 [high, 1.27 (1.14–1.41)], CA199 [high, 1.25 (1.12–1.39)], location [colon, 1.18 (1.07–1.31)] and lung metastasis [yes, 1.15 (1.03–1.29)].

The results of the multivariate Cox proportional hazards regression analysis revealed that the training group and all patients were screened for the same risk factors, indicating that the training group represented all patients well. Moreover, these 11 statistically significant risk factors were also selected in the validation group by univariate Cox proportional hazards regression analysis.

### 3.3 Establishment of the Nomogram Model

**FIGURE 3:**
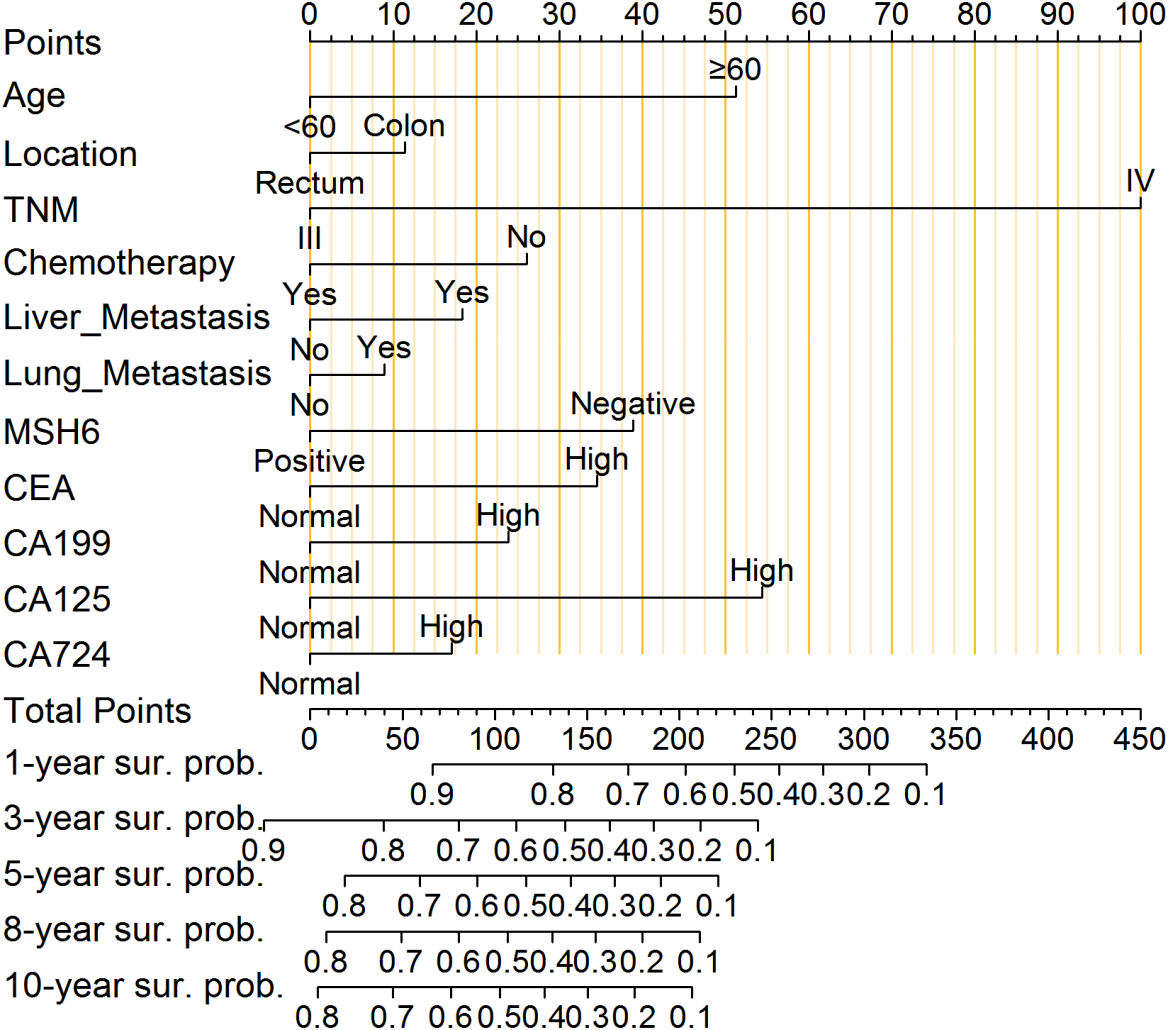
Nomogram for predicting 1‐, 3‐, 5‐, 8‐ and 10‐year overall survival (OS) in patients with advanced stage colorectal cancer (CRC). A straight line is drawn vertically from the axis of each variable to reach the top ‘Points’ scale. The score of the 11 variables are summed and a line is draw vertically from the ‘Total Points’ scale to intercept the survival axes. The total points on the bottom scales that correspond to the 1‐, 3‐, 5‐, 8‐ and 10‐year OS rates.

A nomogram for predicting 1‐, 3‐, 5‐, 8‐ and 10‐year OS in patients with advanced stage CRC was constructed by applying independent risk factors on the basis of the results of multivariate Cox proportional hazards regression analysis in the training cohort, as shown in Figure 3. Each independent risk factor corresponds to a specific score ranging from 0 to 100, which is derived by drawing a straight line on the score scale at the top of the nomogram model, with higher scores indicating a stronger influence of the risk factor. The impact of risk factors was in the following order: TNM (IV), CA125 (high), age (≥ 60), MSH6 (negative), CEA (high), chemotherapy (no), CA199 (high), liver metastasis (yes), CA724 (high), location (colon) and lung metastasis (yes). The total score was obtained by summing the scores for each risk factor, and a vertical line was drawn from the total score axis corresponding to OS at the 1, 3, 5, 8 and 10 years, with higher total scores being associated with lower OS.

For example, a patient was randomly selected from the training group: a 50‐year‐old man (0 points); primary cancer located in the colon (11.25 points); TNM stage III (0 points); chemotherapy (0 points), cancer metastasising to the liver and lung (18.25 and 8.87 points); MSH6 test positive (0 points) and tumour marker CEA, CA199, CA125 and CA724 levels (35.00, 23.75, 54.00 and 17.00 points respectively) that exceeded the normal reference range. Therefore, the cumulative total score for each risk factor was 168, with corresponding 1‐, 3‐, 5‐, 8‐ and 10‐year OS rates of 70.10%, 38.25%, 27.55%, 23.00% and 20.50% respectively. The main risk factors affecting this patient's OS rate were cancer located in the colon, liver and lung cancer metastases and elevated tumour marker levels.

### 3.4 ROC Curves

**FIGURE 4:**
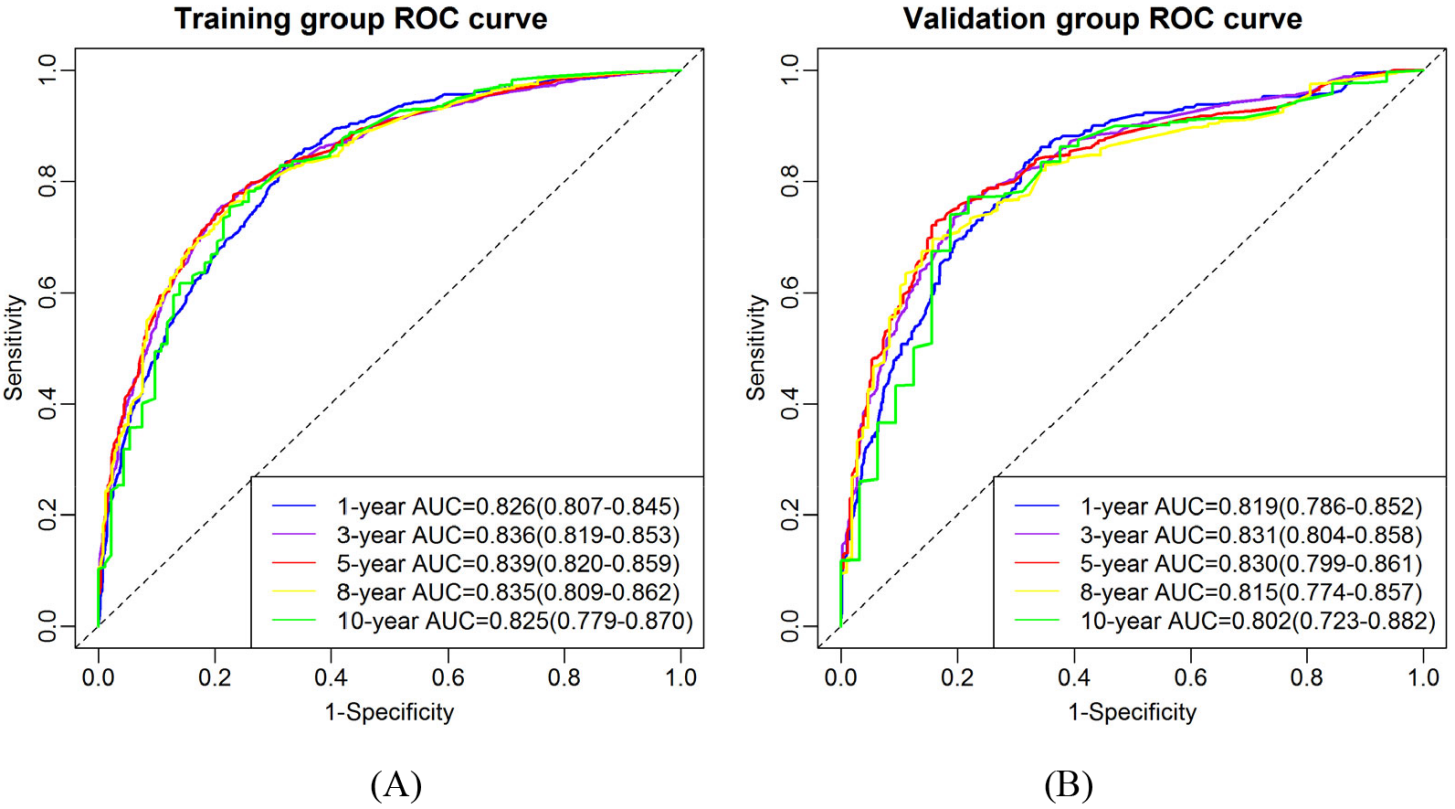
Nomogram predicting receiver operation characteristic (ROC) curves for 1‐, 3‐, 5‐, 8‐ and 10‐year overall survival (OS) in different groups of advanced stage colorectal cancer (CRC) patients. (A) Area under the curve (AUC) values of the ROC curve in the training group. (B) AUC values of the ROC curve in the validation group.

The ROC curves and the AUC values were used to evaluate the discrimination ability of the nomogram. In the training group, the ROC curves (Figure 4A) showed that the established nomogram had good predictive efficacy for 1‐, 3‐, 5‐, 8‐ and 10‐year OS of advanced stage CRC patients, with AUC values of 0.826 (95% CI: 0.807–0.845), 0.836 (0.819–0.853), 0.839 (0.820–0.859), 0.835 (0.809–0.862) and 0.825 (0.779–0.870) respectively. In the validation group, the results of the ROC curves (Figure 4B) were also very satisfactory, with AUC values of 0.819 (95% CI: 0.786–0.852), 0.831 (0.804–0.858), 0.830 (0.799–0.861), 0.815 (0.774–0.857) and 0.802 (0.723–0.882) respectively. The established nomogram was effective in predicting 1‐, 3‐, 5‐, 8‐ and 10‐year OS of advanced stage CRC patients, with AUC values greater than 0.800 and the maximum values at 3 and 5 years greater than 0.830, indicating high discriminatory ability. In addition, the AUC values of the validation and training groups were extremely similar, which indicates that the nomogram model has good predictive ability in both groups.

Finally, we evaluated the performance of 11 risk factors in the prediction of 1‐, 3‐, 5‐, 8‐ and 10‐year OS of advanced stage CRC patients by ROC analysis in the training and validation groups. As shown in Figure S1, the top three risk factors in terms of AUC values were TNM, liver metastasis and CEA, and the combination model was significantly superior to the single risk factor model in predicting the OS of advanced stage CRC patients.

### 3.5 Calibration Curves

**FIGURE 5:**
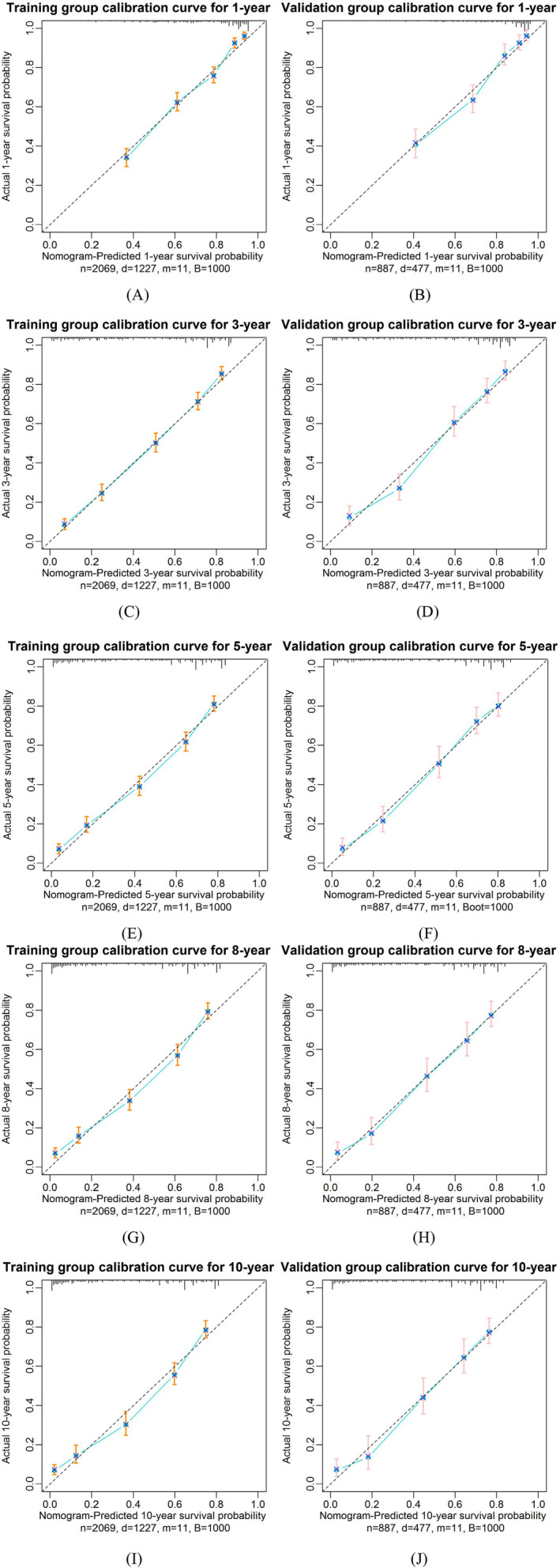
Nomogram predicting calibration curves for 1‐, 3‐, 5‐, 8‐ and 10‐year overall survival (OS) in different groups of advanced stage colorectal cancer (CRC) patients. The diagonal dotted line shows the equality between the actual and predicted OS probabilities, indicating a perfect prediction. The predictive performance of the nomogram is represented by the blue solid lines. The vertical bars indicate 95% confidence intervals. The closer the blue solid line fit is to the diagonal dotted line, the higher the prediction accuracy of the nomogram. n, d, m and B denote the number of patients, the number of cases with a fatal event, the number of independent risk factors and the number of bootstrap samples in the model respectively.

The calibration curves were used to evaluate the consistency between the nomogram‐predicted and actual OS. In the training and validation groups, the calibration curves (Figure 5) revealed that the nomogram‐predicted OS of advanced stage CRC patients at 1, 3, 5, 8 and 10 years were in good agreement with the actual OS. The calibration curves predicted by the nomogram model almost coincided with the ideal diagonal dotted line and were all within the 95% CIs of the calibration curves, indicating that the model had good calibration ability.

### 3.6 DCA Curves

**FIGURE 6:**
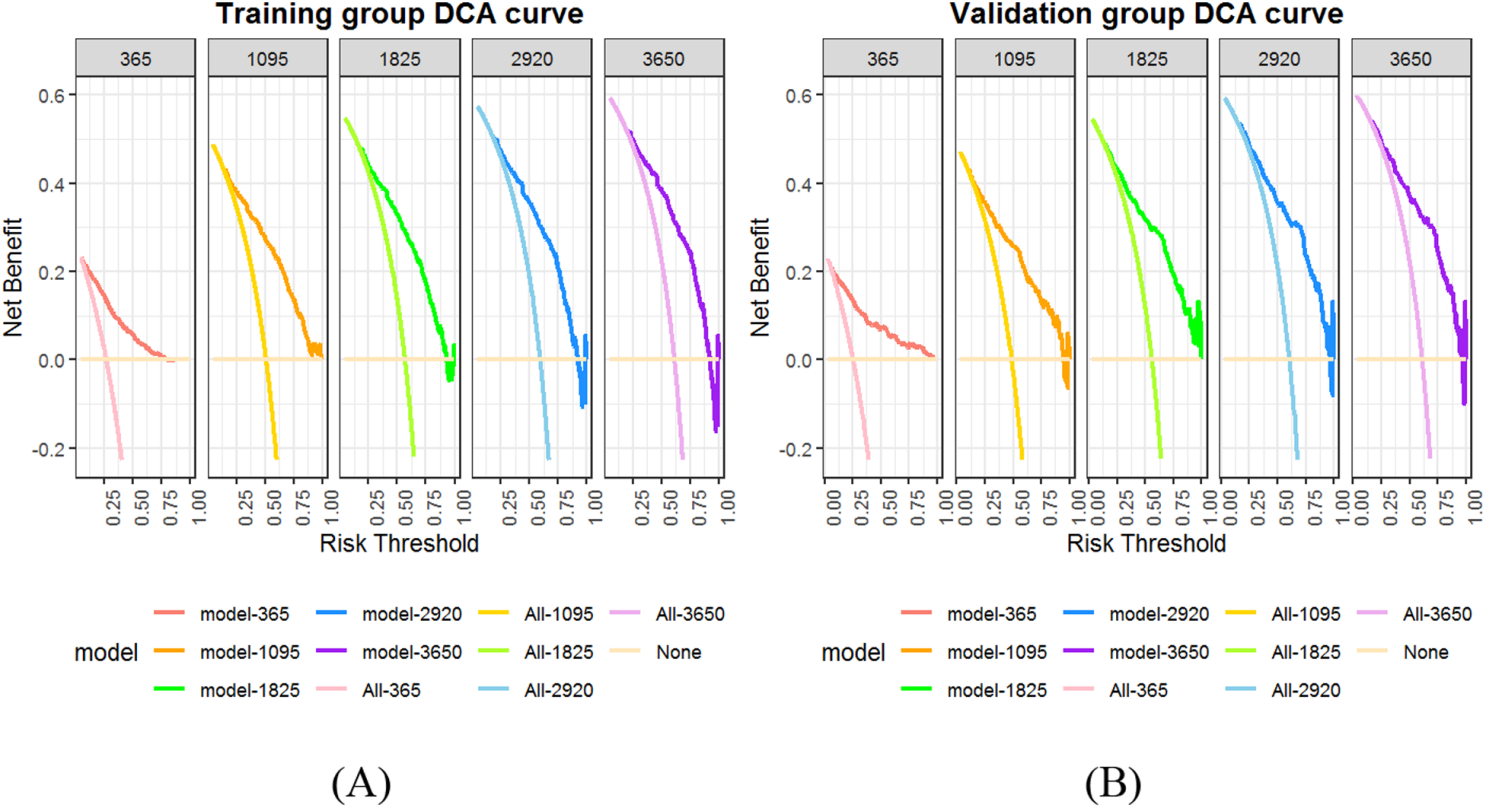
Nomogram predicting decision curve analysis (DCA) curves for 1‐, 3‐, 5‐, 8‐ and 10‐year overall survival (OS) in different groups of advanced stage colorectal cancer (CRC) patients. The *x*‐axis represents the risk threshold and the *y*‐axis represents the net benefit. The horizontal line represents no patients undergoing the clinical intervention (all patients survived), the middle light‐coloured line indicates that all patients implemented the clinical intervention (all patients died) and the top dark‐coloured line is the DCA curve predicted by the nomogram model.

To further evaluate the clinical utility value of the nomogram, DCA for 1‐, 3‐, 5‐, 8‐ and 10‐year OS was performed for advanced stage CRC patients, as shown in Figure 6. The range of risk thresholds corresponding to the DCA curve above the other two lines is considered to be the model's ability to predict the net benefit from implementing the clinical intervention. The results showed that in the training group, the risk threshold for the model predicting benefit from implementing clinical interventions for OS at 1 year ranged from 0.10 to 0.75 and those at 3, 5, 8 and 10 years were approximately 0.20–0.90. Similarly, in the validation group, the risk threshold for benefit at 1 year ranged from 0.10 to 0.90 and those at 3, 5, 8 and 10 years were approximately 0.20–0.95. As the risk threshold increases, the model predicts a decreasing trend in the net benefit exhibited by the DCA curve. Moreover, the DCA curve revealed that the predictive nomogram had a high net benefit, which implies that it has good clinical implementation implications.

### 3.7 OS Rates for Different Strata of Risk Factors

In this study, the OS rates at 1, 3, 5, 8 and 10 years for all patients with advanced stage CRC were 73.4 (95% CI: 71.8–75.0), 49.5 (47.8–51.4), 43.3 (41.5–45.2), 40.1 (38.1–41.9) and 38.6 (36.6–40.8) respectively. The 10‐year Kaplan–Meier curves were plotted for different strata of 11 risk factors in advanced stage CRC patients (Figure S2) and the specific OS rates were presented in Table S2.

## 4 Discussion

CRC is obviously diversified with regard to patient survival, even when all of the patients go through surgical resection [23, 30]. Additional individualised treatment may offer improved OS in high‐risk patients with CRC. Therefore, constructing a prognostic model to determine the risk factors and prognostic outcomes that affect postoperative OS in CRC patients is important for the development of clinical treatment plans. Currently, an ideal survival prediction model developed for advanced stage CRC has not yet been created. In this study, we constructed a nomogram model to predict the risk of 1‐, 3‐, 5‐, 8‐ and 10‐year OS after surgery in advanced stage CRC patients and validated the performance of the model. Effective clinical interventions are proposed to improve postoperative OS in conjunction with meaningful influencing factors.

Accurate calculation of 1‐, 3‐, 5‐, 8‐ and 10‐year postoperative OS of advanced stage CRC patients may provide a reference for knowing the progression of the disease after surgery in advance. The OS of advanced stage CRC patients in this study was lower than that of early‐stage (stages I–II) and all patients (stages I–IV), but higher than that of stage IV patients [20, 31, 32, 33]. This is mainly due to the fact that early‐stage CRC patients are primary cancers without metastasis and have a better prognosis after surgical resection [34]. However, stage IV CRC patients have metastasised to other sites or organs, especially multiple metastases, resulting in organ decompensation or failure, unsatisfactory surgical results and prognosis and relatively high risk of death in the short term [35]. Liver and lung metastases are the most common secondary malignancies in CRC patients and their OS is significantly lower than that of patients without metastases [35, 36]. The results of this study confirm this view. CRC patients with combined liver and lung metastases have significantly lower OS than those with single lung or liver metastases [37].

Nomograms are better than traditional TNM staging systems in assessing the prognosis of cancer and they provide more informative quantitative estimates of the probability of OS and the impact of each risk factor than other models [27, 38]. Several studies have applied nomogram models to analyse the OS of CRC patients and used AUC values to evaluate the discrimination of the model [39, 40, 41]. The AUC values calculated in this study are significantly greater than those reported by the aforementioned scholars, which suggests that the constructed nomogram model provided good discriminatory capacity. The AUC values of the model prediction training and validation groups are both greater than 0.8 with small errors, indicating that the nomogram model has good prediction performance, and the internal dataset validation is applicable without overfitting. The predictive effect of the nomogram may be confounded by certain factors. It has been shown that the nomogram model constructed based on the training group predicts the internal validation group well, but the external validation group is poorly predicted, as evidenced by the AUC values of the external validation group is significantly lower than that of the training group and the internal validation group [33, 42]. We attribute this result to the fact that the applicability of the nomogram is not entirely consistent across populations or the external validation sample size is insufficient and has not yet reached a steady state.

Several clinical characteristics were proven to be independent prognostic factors for OS in this study, including age, TNM stage, location, liver and lung metastases, chemotherapy, MSH6, CEA, CA125, CA199 and CA724 levels. Most of these risk factors are consistent with those previously reported. Many studies have shown that advanced age is associated with a low survival probability in patients with CRC, but there is no clear age stratification criterion [20, 33, 35, 43]. In this study, we used 60 years as the age threshold and therefore estimated age (≥ 60, HR = 1.85). Currently, the TNM stage is used to predict the outcome of postoperative CRC patients, with a higher TNM stage corresponds to a lower survival probability [21, 28]. Our results are consistent with this finding and estimate that TNM (IV, HR = 3.15).

The effect of tumour location on the OS in CRC patients is more controversial, with some scholars suggesting that tumour location has no effect on OS [21, 25, 27, 39]. However, some studies have reported that tumour location in the colon or rectum corresponds to a higher risk of low OS and the results of the current studies are inconsistent [28, 44, 45]. This study also revealed that a tumour location in the colon (colon, HR = 1.14) was associated with a high risk of low OS. Chemotherapy is a common and effective regimen for the treatment of CRC [21, 22, 33]. Our findings support the conclusion that patients who did not receive chemotherapy (no, HR = 1.30) had a significantly lower OS.

Numerous studies have shown that organ metastases of CRC patients significantly contribute to increased mortality and poor prognosis [21, 26, 33]. We verified that the occurrence of liver and lung metastases in advanced stage CRC patients significantly increased the risk of low OS for liver metastasis (yes, HR = 1.30), lung metastasis (yes, HR = 1.15) respectively. Controversially, Yu and Zhang [27] and Wu et al. [28] revealed that bone metastasis and brain metastasis were independent prognostic factors for CRC patients; however, this finding was not confirmed in the advanced stage CRC patients in this study, which may be due to the differences in the study cohort or the small number of patients who developed bone and brain metastasis, which needs to be verified by further increasing the sample size.

Recently, serum biomarkers have been widely used for the clinical diagnosis, postoperative monitoring and prognosis of CRC patients [46]. Tumour biomarkers CEA, CA199, CA242, CA125, CA50 and CA724 play important roles in monitoring recurrence and metastasis in patients with CRC, and in particular, CEA and CA199 levels were significantly associated with the prognosis of CRC patients [25, 26, 47]. Similar to the earlier results, we estimated that the serum markers CEA, CA199, CA125 and CA724 were independent risk factors for OS in advanced stage CRC patients.

As one of the three major mechanisms of CRC carcinogenesis, MSI occurs in approximately 15% of CRC cases [48]. The occurrence of MSI is associated with mutations in one of genes MLH1, MSH2, MSH6 or PMS2 [49]. The value of MSI in CRC diagnosis, treatment response and prognosis has attracted interest [48, 50]. MSI has been validated as a key factor influencing the prognosis of CRC, especially in early‐stage cases [51, 52]. In contrast, Guo et al. [26] reported that mutations in the MLH1, MSH2, MSH6 or PMS2 genes were not associated with OS of CRC patients, possibly because of the insufficient sample size. Our study confirmed that mutation of the MSH6 gene (negative, HR = 1.51) is a risk factor of OS in patients with advanced stage CRC and is associated with CRC prognosis.

Limitations of this study include the single‐centre design and the lack of analysis of cancer‐specific survival. In addition, some prognostic factors could not be included because of the retrospective design. In future studies, external validation will be performed by using data from multiple institutions.

## 5 Conclusion

In summary, we constructed and validated an original predictive nomogram that can accurately predict the OS of advanced stage CRC patients. Moreover, our nomogram was demonstrated to have high accuracy and reliability through the validation of discrimination and calibration and to perform well in terms of clinical utility.

## Author Contributions

**Pengwei Lou and Dongmei Luo:** conceived and designed the experiments. **Pengwei Lou and Kai Wang:** methodology and data curation. **Pengwei Lou, Dongmei Luo and Kai Wang:** software. **Dongmei Luo and Yuting Huang:** validation. **Pengwei Lou, Yuting Huang and Shuai Yuan:** formal analysis. **Dongmei Luo and Chen Chen:** investigation. **Dongmei Luo and Shuai Yuan:** resources. **Pengwei Lou and Yuting Huang:** writing – original draft. **Shuai Yuan and Kai Wang:** supervision and writing – review and editing. **Pengwei Lou, Shuai Yuan and Kai Wang:** visualisation. **Kai Wang:** project administration. All authors critically read the manuscript and gave final approval for publication.

## Ethics Statement

This study was approved by the institutional ethics committee of the Cancer Hospital Affiliated with Xinjiang Medical University (IRB No. K‐2023005), and all participating patients provided informed consent.

## Conflicts of Interest

The authors declare no conflicts of interest.

## Supporting information


**TABLE S1.** Univariate Cox proportional hazards regression analysis of overall survival (OS) in different groups of patients with advanced stage colorectal cancer (CRC).

**TABLE S2.** Survival probability at 1, 3, 5, 8 and 10 years for different strata of risk factors in patients with advanced stage colorectal cancer (CRC).

**FIGURE S1.** Nomogram predicting receiver operating characteristic (ROC) curves for 1‐, 3‐, 5‐, 8‐ and 10‐year overall survival (OS) in different groups of advanced stage colorectal cancer (CRC) patients. (A–J) Area under the curve (AUC) values of the ROC curve for the 11 risk factors in the training and validation groups.

**FIGURE S2.** Ten‐year overall survival (OS) Kaplan–Meier curves for advanced stage colorectal cancer (CRC) patients stratified by different risk factors.

## Data Availability

The original database containing confidential patient information cannot be made public. The anonymised data that were used in this study are available via reasonable request to the corresponding authors.
