# Supplementary material for: Establishment and Validation of a Prognostic Nomogram for Predicting Postoperative Overall Survival in Advanced Stage III–IV Colorectal Cancer Patients
Source: Cancer Med. 2024 Nov 15;13(22):e70385. doi: 10.1002/cam4.70385 (PMC11566917; doi:10.1002/cam4.70385)
Supplement: Supplementary file 1 — TABLE S1. Univariate Cox proportional hazards regression analysis of overall survival (OS) in different groups of patients with advanced stage colorectal cancer (CRC). [file CAM4-13-e70385-s001.docx]

**TABLE S1** Univariate Cox proportional hazards regression analysis of OS in different groups of patients with advanced CRC

| **Variables** | **Training group** | |  | **Validation group** | |  | **All patients** |  |
| --- | --- | --- | --- | --- | --- | --- | --- | --- |
|  | **HR (95% CI)** | ***P*** |  | **HR (95% CI)** | ***P*** |  | **HR (95% CI)** | ***P*** |
| Age |  |  |  |  |  |  |  |  |
| <60 | Reference |  |  | Reference |  |  | Reference |  |
| ≥60 | 1.59 (1.42-1.78) | < 0.001 |  | 1.27 (1.06-1.52) | 0.010 |  | 1.48 (1.35-1.63) | < 0.001 |
| Gender |  |  |  |  |  |  |  |  |
| Female | Reference |  |  | Reference |  |  | Reference |  |
| Male | 1.07 (0.96-1.20) | 0.223 |  | 1.06 (0.88-1.27) | 0.530 |  | 1.07 (0.97-1.18) | 0.180 |
| Location |  |  |  |  |  |  |  |  |
| Rectum | Reference |  |  | Reference |  |  | Reference |  |
| Colon | 1.14 (1.02-1.28) | 0.020 |  | 1.11 (0.92-1.32) | 0.028 |  | 1.13 (1.03-1.25) | 0.010 |
| TNM |  |  |  |  |  |  |  |  |
| III | Reference |  |  | Reference |  |  | Reference |  |
| IV | 4.68 (4.13-5.31) | < 0.001 |  | 4.70 (3.86-5.72) | < 0.001 |  | 4.70 (4.23-5.23) | < 0.001 |
| T |  |  |  |  |  |  |  |  |
| T2 | Reference |  |  | Reference |  |  | Reference |  |
| T3 | 1.03 (0.77-1.38) | 0.844 |  | 0.98 (0.61-1.59) | 0.948 |  | 1.02 (0.79-1.30) | 0.905 |
| T4 | 1.48 (1.11-1.97) | 0.007 |  | 1.27 (0.80-2.01) | 0.321 |  | 1.41 (1.11-1.80) | 0.006 |
| N |  |  |  |  |  |  |  |  |
| N0 | Reference |  |  | Reference |  |  | Reference |  |
| N1-2 | 0.74 (0.61-0.90) | 0.002 |  | 0.60 (0.44-0.81) | < 0.001 |  | 0.70 (0.59-0.82) | < 0.001 |
| N3-4 | 1.290 (1.00-1.65) | 0.047 |  | 1.33 (0.92-1.93) | 0.131 |  | 1.30 (1.06-1.60) | 0.012 |
| M |  |  |  |  |  |  |  |  |
| M0 | Reference |  |  | Reference |  |  | Reference |  |
| M1 | 1.93 (1.69-2.21) | < 0.001 |  | 2.04 (1.65-2.51) | < 0.001 |  | 1.97 (1.76-2.20) | < 0.001 |
| Radiotherapy |  |  |  |  |  |  |  |  |
| No | Reference |  |  | Reference |  |  | Reference |  |
| Yes | 1.06 (0.95-1.19) | 0.292 |  | 0.89 (0.74-1.07) | 0.201 |  | 1.01 (0.92-1.12) | 0.776 |
| Chemotherapy |  |  |  |  |  |  |  |  |
| Yes | Reference |  |  | Reference |  |  | Reference |  |
| No | 1.48 (1.24-1.77) | < 0.001 |  | 1.48 (1.07-2.04) | 0.017 |  | 1.49 (1.28-1.74) | < 0.001 |
| Liver metastasis |  |  |  |  |  |  |  |  |
| No | Reference |  |  | Reference |  |  | Reference |  |
| Yes | 2.87 (2.56-3.22) | < 0.001 |  | 2.98 (2.48-3.58) | < 0.001 |  | 2.91 (2.64-3.20) | < 0.001 |
| Lung metastasis |  |  |  |  |  |  |  |  |
| No | Reference |  |  | Reference |  |  | Reference |  |
| Yes | 1.86 (1.65-2.10) | < 0.001 |  | 1.97 (1.62-2.41) | < 0.001 |  | 1.90 (1.71-2.11) | < 0.001 |
| Bone metastasis |  |  |  |  |  |  |  |  |
| No | Reference |  |  | Reference |  |  | Reference |  |
| Yes | 1.06 (0.92-1.23) | 0.425 |  | 1.26 (0.99-1.61) | 0.066 |  | 1.14 (0.98-1.40) | 0.176 |
| Splenic metastasis |  |  |  |  |  |  |  |  |
| No | Reference |  |  | Reference |  |  | Reference |  |
| Yes | 1.33 (0.50-3.55) | 0.568 |  | 1.82 (0.59-5.67) | 0.300 |  | 1.26 (0.63-2.53) | 0.509 |
| Metastasis to other  body parts |  |  |  |  |  |  |  |  |
| No | Reference |  |  | Reference |  |  | Reference |  |
| Yes | 0.99 (0.88-1.11) | 0.840 |  | 0.95 (0.79-1.15) | 0.579 |  | 0.98 (0.88-1.08) | 0.633 |
| Diameters |  |  |  |  |  |  |  |  |
| <5.5 | Reference |  |  | Reference |  |  | Reference |  |
| ≥5.5 | 0.94 (0.84-1.05) | 0.262 |  | 0.87 (0.73-1.05) | 0.148 |  | 0.92 (0.84-1.01) | 0.086 |
| Tumour differentiation |  |  |  |  |  |  |  |  |
| High | Reference |  |  | Reference |  |  | Reference |  |
| Middle | 0.77 (0.63-0.93) | 0.007 |  | 0.76 (0.54-1.06) | 0.102 |  | 0.76 (0.65-0.90) | 0.001 |
| Low | 1.19 (0.95-1.49) | 0.129 |  | 0.89 (0.60-1.31) | 0.554 |  | 1.09 (0.90-1.32) | 0.396 |
| Nerve invasion |  |  |  |  |  |  |  |  |
| No | Reference |  |  | Reference |  |  | Reference |  |
| Yes | 0.97 (0.86-1.10) | 0.650 |  | 0.95 (0.78-1.16) | 0.635 |  | 0.97 (0.87-1.07) | 0.515 |
| Vascular invasion |  |  |  |  |  |  |  |  |
| No | Reference |  |  | Reference |  |  | Reference |  |
| Yes | 1.04 (0.92-1.17) | 0.524 |  | 0.96 (0.79-1.17) | 0.680 |  | 1.02 (0.92-1.13) | 0.752 |
| Extramural vascular invasion |  |  |  |  |  |  |  |  |
| No | Reference |  |  | Reference |  |  | Reference |  |
| Yes | 1.06 (0.82-1.38) | 0.655 |  | 1.09 (0.69-1.73) | 0.714 |  | 1.08 (0.86-1.35) | 0.519 |
| Lymph node metastasis |  |  |  |  |  |  |  |  |
| No | Reference |  |  | Reference |  |  | Reference |  |
| Yes | 0.86 (0.70-1.07) | 0.169 |  | 0.82 (0.58-1.17) | 0.277 |  | 0.85 (0.71-1.02) | 0.074 |
| Surgical method |  |  |  |  |  |  |  |  |
| Palliative resection | Reference |  |  | Reference |  |  | Reference |  |
| Radical resection | 1.15 (0.92-1.43) | 0.223 |  | 0.90 (0.64-1.25) | 0.528 |  | 1.07 (0.89-1.28) | 0.487 |
| Enlarging resection | 1.13 (0.82-1.58) | 0.459 |  | 1.13 (0.68-1.89) | 0.637 |  | 1.13 (0.86-1.49) | 0.385 |
| Circumferential margin |  |  |  |  |  |  |  |  |
| No | Reference |  |  | Reference |  |  | Reference |  |
| Yes | 1.10 (0.88-1.38) | 0.397 |  | 1.35 (0.97-1.89) | 0.078 |  | 1.17 (0.97-1.41) | 0.102 |
| MLH1 |  |  |  |  |  |  |  |  |
| Positive | Reference |  |  | Reference |  |  | Reference |  |
| Negative | 1.11 (0.77-1.35) | 0.894 |  | 0.98 (0.62-1.53) | 0.920 |  | 1.01(0.80-1.28) | 0.947 |
| MSH2 |  |  |  |  |  |  |  |  |
| Positive | Reference |  |  | Reference |  |  | Reference |  |
| Negative | 0.99 (0.70-1.39) | 0.937 |  | 1.01 (0.52-1.94) | 0.989 |  | 1.00 (0.74-1.36) | 0.997 |
| MSH6 |  |  |  |  |  |  |  |  |
| Positive | Reference |  |  | Reference |  |  | Reference |  |
| Negative | 1.39 (1.01-1.93) | 0.044 |  | 1.54 (0.92-2.58) | 0.048 |  | 1.44 (1.09-1.89) | 0.009 |
| PMS2 |  |  |  |  |  |  |  |  |
| Positive | Reference |  |  | Reference |  |  | Reference |  |
| Negative | 1.19 (0.88-1.62) | 0.259 |  | 0.90 (0.53-1.53) | 0.702 |  | 1.10 (0.85-1.44) | 0.464 |
| Microsatellite |  |  |  |  |  |  |  |  |
| Stability | Reference |  |  | Reference |  |  | Reference |  |
| Low instability | 0.98 (0.76-1.28) | 0.898 |  | 1.11 (0.71-1.74) | 0.651 |  | 1.02 (0.81-1.28) | 0.868 |
| Highly unstable | 1.22 (0.91-1.62) | 0.187 |  | 0.98 (0.60-1.62) | 0.949 |  | 1.15 (0.89-1.47) | 0.278 |
| CEA |  |  |  |  |  |  |  |  |
| Normal | Reference |  |  | Reference |  |  | Reference |  |
| High | 2.46 (2.18-2.78) | < 0.001 |  | 2.53 (2.09-3.08) | < 0.001 |  | 2.49 (2.24-2.76) | < 0.001 |
| CA199 |  |  |  |  |  |  |  |  |
| Normal | Reference |  |  | Reference |  |  | Reference |  |
| High | 2.34 (2.09-2.62) | < 0.001 |  | 2.37 (1.97-2.85) | < 0.001 |  | 2.36 (2.14-2.60) | < 0.001 |
| CA125 |  |  |  |  |  |  |  |  |
| Normal | Reference |  |  | Reference |  |  | Reference |  |
| High | 2.71 (2.39-3.08) | < 0.001 |  | 3.03 (2.48-3.71) | < 0.001 |  | 2.80 (2.51-3.12) | < 0.001 |
| AFP |  |  |  |  |  |  |  |  |
| Normal | Reference |  |  | Reference |  |  | Reference |  |
| High | 2.31 (1.20-4.46) | 0.012 |  | 1.26 (0.47-3.36) | 0.649 |  | 1.80 (1.04-3.11) | 0.035 |
| CA724 |  |  |  |  |  |  |  |  |
| Normal | Reference |  |  | Reference |  |  | Reference |  |
| High | 2.06 (1.84-2.31) | < 0.001 |  | 2.13 (1.77-2.55) | < 0.001 |  | 2.08 (1.89-2.29) | < 0.001 |

Abbreviations: 95% CI, 95% confidence interval; HR, hazard ratio.
