# Supplementary material for: Establishment and Validation of a Prognostic Nomogram for Predicting Postoperative Overall Survival in Advanced Stage III–IV Colorectal Cancer Patients
Source: Cancer Med. 2024 Nov 15;13(22):e70385. doi: 10.1002/cam4.70385 (PMC11566917; doi:10.1002/cam4.70385)
Supplement: Supplementary file 2 — TABLE S2. Survival probability at 1, 3, 5, 8 and 10 years for different strata of risk factors in patients with advanced stage colorectal cancer (CRC). [file CAM4-13-e70385-s002.docx]

**TABLE S2** Survival probability at 1, 3, 5, 8, and 10 years for different strata of risk factors in patients with advanced CRC.

| **Variables** | | **1 year** | **3 years** | **5 years** | **8 years** | **10 years** |
| --- | --- | --- | --- | --- | --- | --- |
| All patients |  | 73.4 (71.8-75.0) | 49.5 (47.8-51.4) | 43.3 (41.5-45.2) | 40.1 (38.1-41.9) | 38.6 (36.6-40.8) |
| Age | <60 | 78.3 (76.1-80.5) | 56.5 (53.9-59.1) | 50.3 (47.7-53.0) | 47.5 (44.8-50.4) | 46.7 (43.8-49.8) |
|  | ≥60 | 69.0 (66.8-71.4) | 43.2 (40.8-45.8) | 36.8 (34.4-39.4) | 33.2 (30.7-35.9) | 31.2 (28.4-34.3) |
| TNM | III | 91.9 (90.5-93.3) | 74.8 (72.6-77.0) | 67.4 (65.1-69.9) | 63.8 (61.1-66.5) | 62.4 (59.5-65.4) |
|  | IV | 54.4 (51.9-57.0) | 23.5 (21.4-25.8) | 17.9 (16.0-20.1) | 15.2 (13.2-17.4) | 14.0 (11.9-16.6) |
| Chemotherapy | Yes | 75.3 (73.7-77.0) | 50.7 (48.9-52.7) | 44.0 (42.1-46.0) | 40.7 (38.7-42.7) | 39.4 (37.2-41.6) |
|  | No | 54.1 (48.5-60.4) | 37.3 (32.0-43.6) | 34.8 (29.6-41.1) | 33.1 (27.7-39.6) | 31.4 (25.5-38.6) |
| Location | Rectum | 77.9 (75.9-80.0) | 51.9 (49.5-54.4) | 44.9 (42.4-47.4) | 40.1 (37.6-42.9) | 37.6 (34.6-40.8) |
|  | Colon | 68.0 (65.6-70.5) | 46.7 (44.1-49.4) | 41.2 (38.6-44.0) | 39.9 (37.3-42.8) | 39.2 (36.9-42.4) |
| Liver metastasis | No | 82.2 (80.5-83.9) | 63.1 (61.0-65.3) | 56.4 (54.2-58.7) | 52.4 (50.0-54.9) | 51.4 (48.8-54.1) |
|  | Yes | 57.3 (54.3-60.3) | 24.5 (22.0-27.3) | 18.5 (16.2-21.2) | 16.8 (14.4-19.6) | 14.5 (11.5-18.2) |
| Lung metastasis | No | 75.4 (73.7-77.2) | 54.7 (52.7-56.8) | 49.1 (47.0-51.2) | 46.0 (43.9-48.3) | 45.0 (42.7-47.5) |
|  | Yes | 66.4 (62.9-70.0) | 31.9 (28.5-35.6) | 23.0 (20.0-26.6) | 19.3 (16.2-23.0) | 16.4 (12.7-21.1) |
| MSH6 | Positive | 73.6 (72.0-75.2) | 49.8 (48.1-51.7) | 43.6 (41.8-45.5) | 40.4 (38.5-42.4) | 39.2 (37.1-41.4) |
|  | Negative | 66.2 (56.1-78.2) | 36.6 (27.0-49.7) | 27.5 (18.7-40.6) | 22.9 (14.2-35.2) | 19.4 (10.4-36.1) |
| CEA | Normal | 85.7 (83.8-87.7) | 67.6 (65.1-70.2) | 60.2 (57.5-63.0) | 56.4 (53.5-59.5) | 55.5 (52.4-58.8) |
|  | High | 63.7 (61.5-66.1) | 35.4 (33.1-37.7) | 30.0 (27.8-32.3) | 27.1 (24.9-29.5) | 25.5 (23.0-28.2) |
| CA199 | Normal | 81.7 (80.0-83.4) | 59.7 (57.6-61.9) | 52.2 (50.0-54.5) | 47.8 (45.5-50.3) | 45.9 (43.2-48.7) |
|  | High | 55.4 (52.3-58.7) | 27.6 (24.9-30.6) | 23.7 (21.1-26.7) | 22.1 (19.5-25.0) | 20.6 (18.0-23.6) |
| CA125 | Normal | 80.7 (79.1-82.3) | 56.0 (54.1-58.1) | 49.0 (46.9-51.1) | 45.2 (43.1-47.5) | 43.8 (41.5-46.3) |
|  | High | 42.9 (39.1-47.2) | 22.5 (19.3-26.2) | 19.0 (16.0-22.6) | 17.9 (15.0-21.6) | 16.8 (13.5-21.1) |
| CA724 | Normal | 81.8 (80.1-83.5) | 58.5 (56.3-60.7) | 51.2 (49.0-53.6) | 47.1 (44.7-49.6) | 45.2 (42.4-48.1) |
|  | High | 57.1 (54.2-60.3) | 32.3 (29.5-35.3) | 27.7 (25.0-30.7) | 26.3 (23.6-29.4) | 25.9 (23.1-29.0) |
