# Supplementary material for: Establishment and Validation of a Prognostic Nomogram for Predicting Postoperative Overall Survival in Advanced Stage III–IV Colorectal Cancer Patients
Source: Cancer Med. 2024 Nov 15;13(22):e70385. doi: 10.1002/cam4.70385 (PMC11566917; doi:10.1002/cam4.70385)
Supplement: Supplementary file 3 — FIGURE S1. Nomogram predicting receiver operating characteristic (ROC) curves for 1‐, 3‐, 5‐, 8‐ and 10‐year overall survival (OS) in different groups of advanced stage colorectal cancer (CRC) patients. (A–J) Area under the curve (AUC) values of the ROC curve for the 11 risk factors in the training and validation groups. [file CAM4-13-e70385-s004.docx]

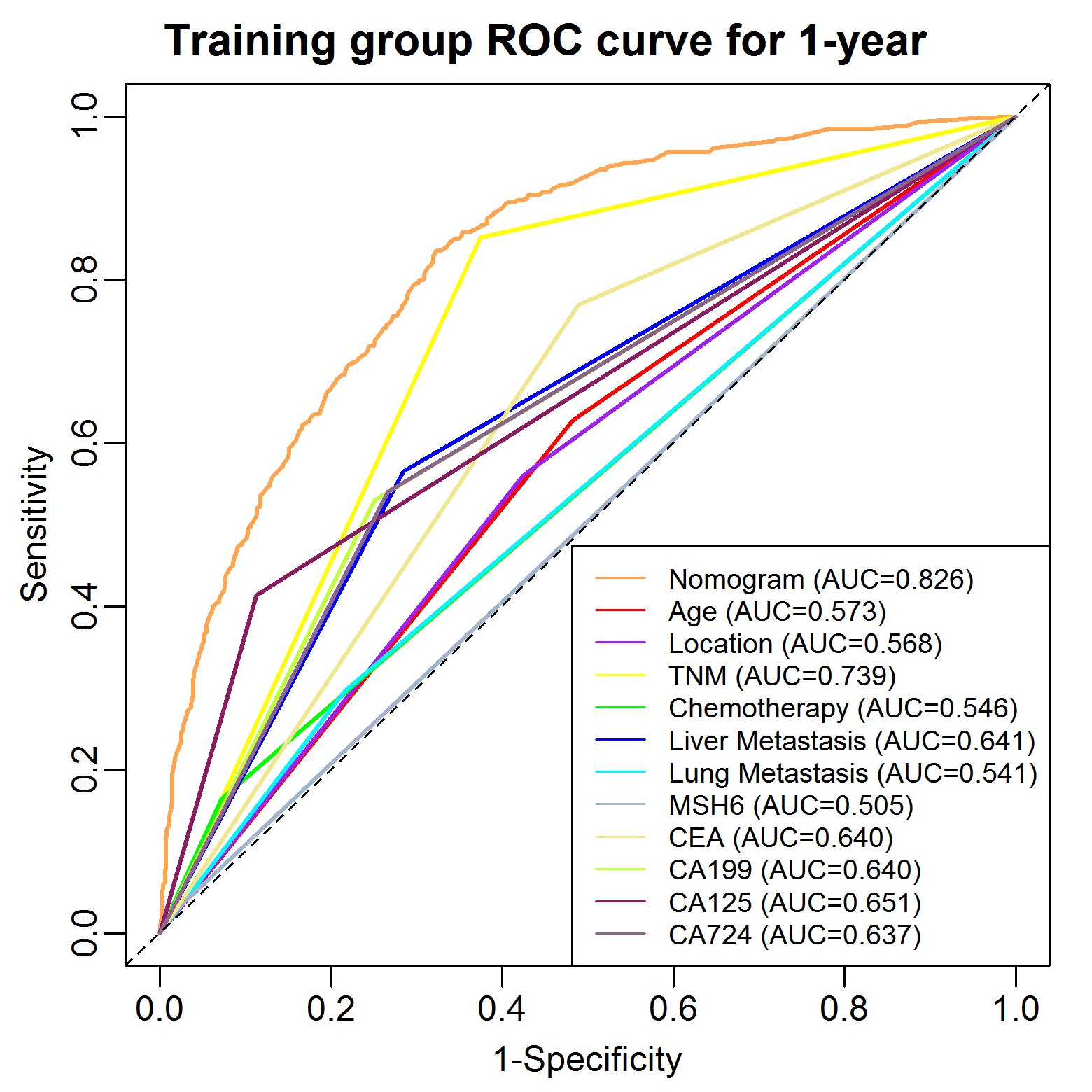

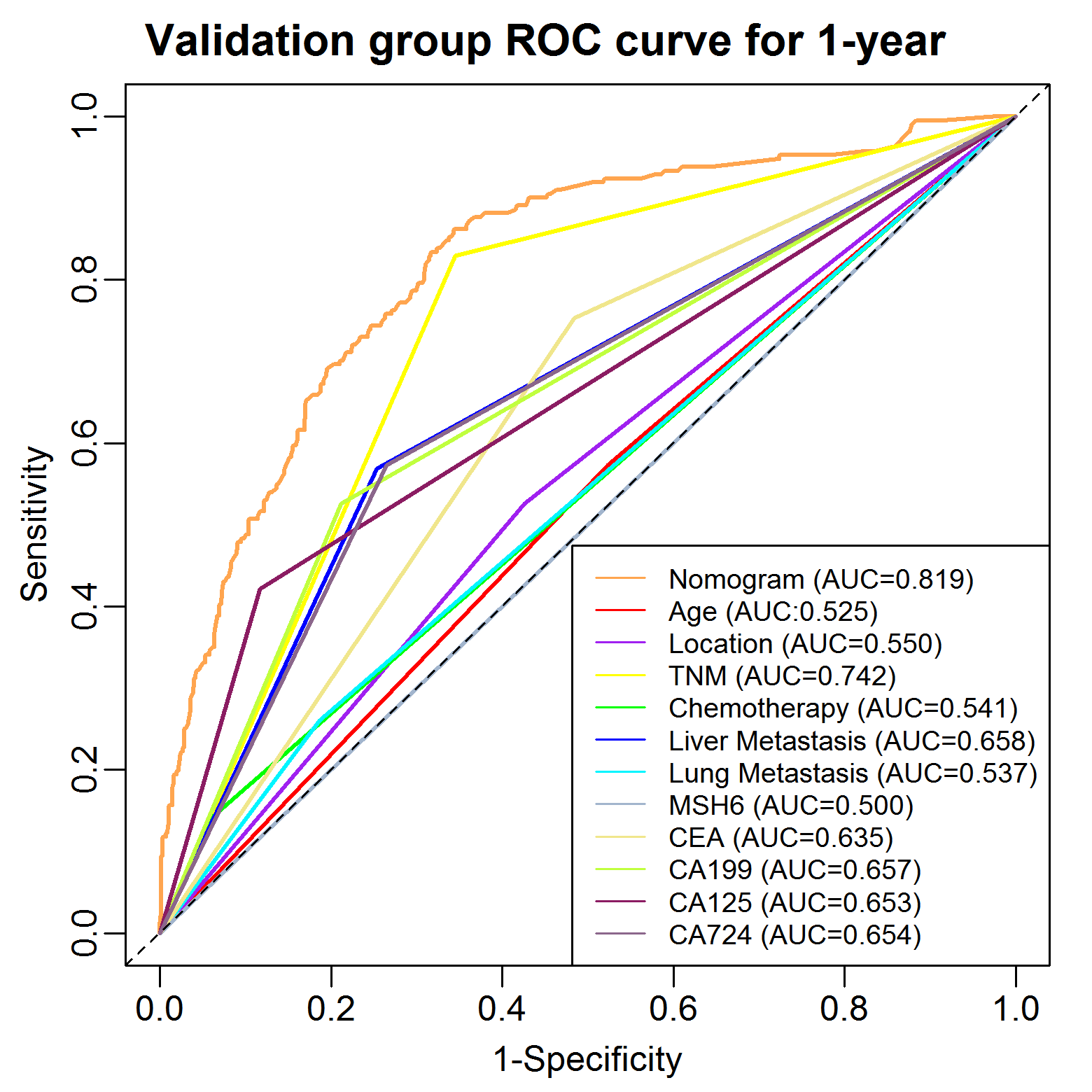


(A) (B)


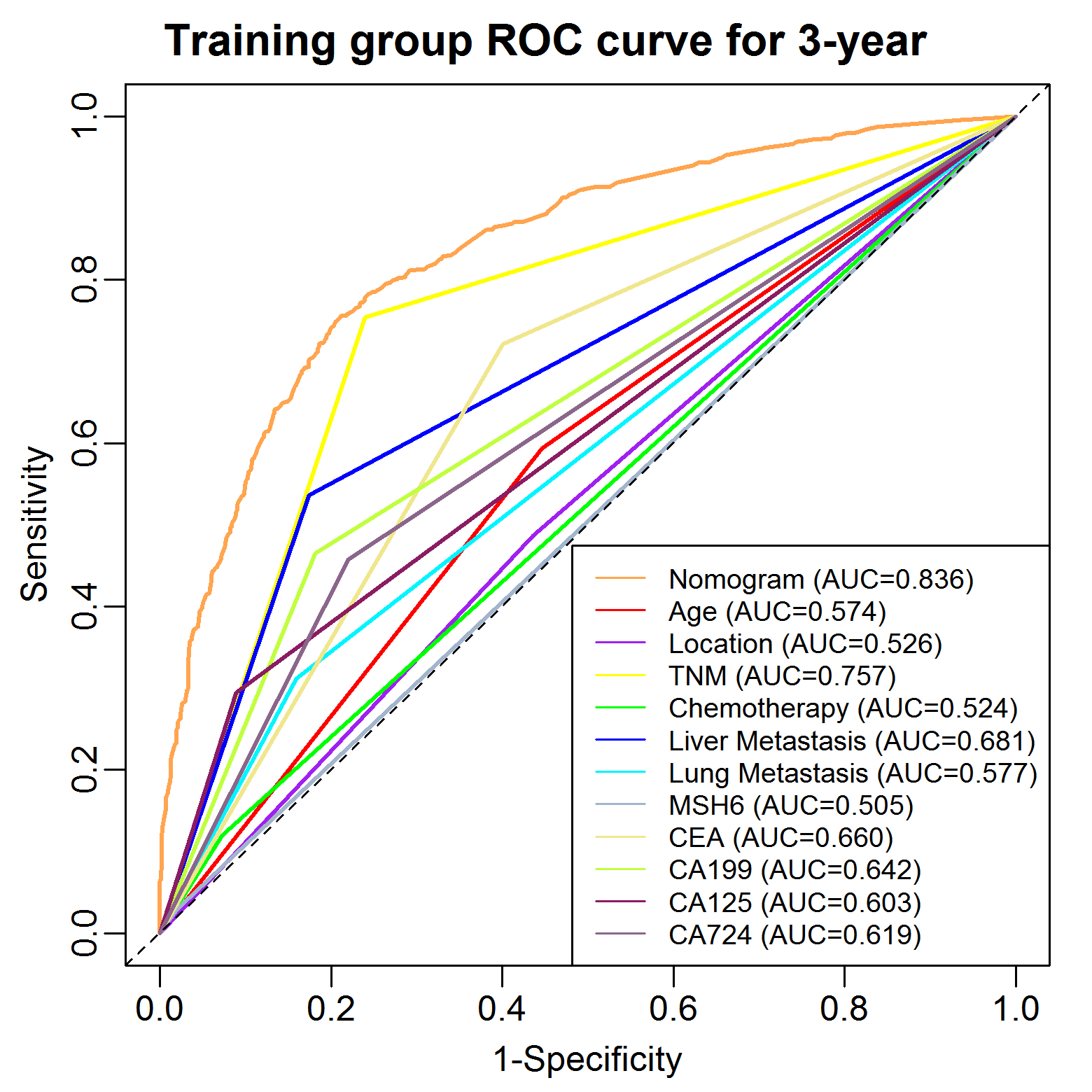

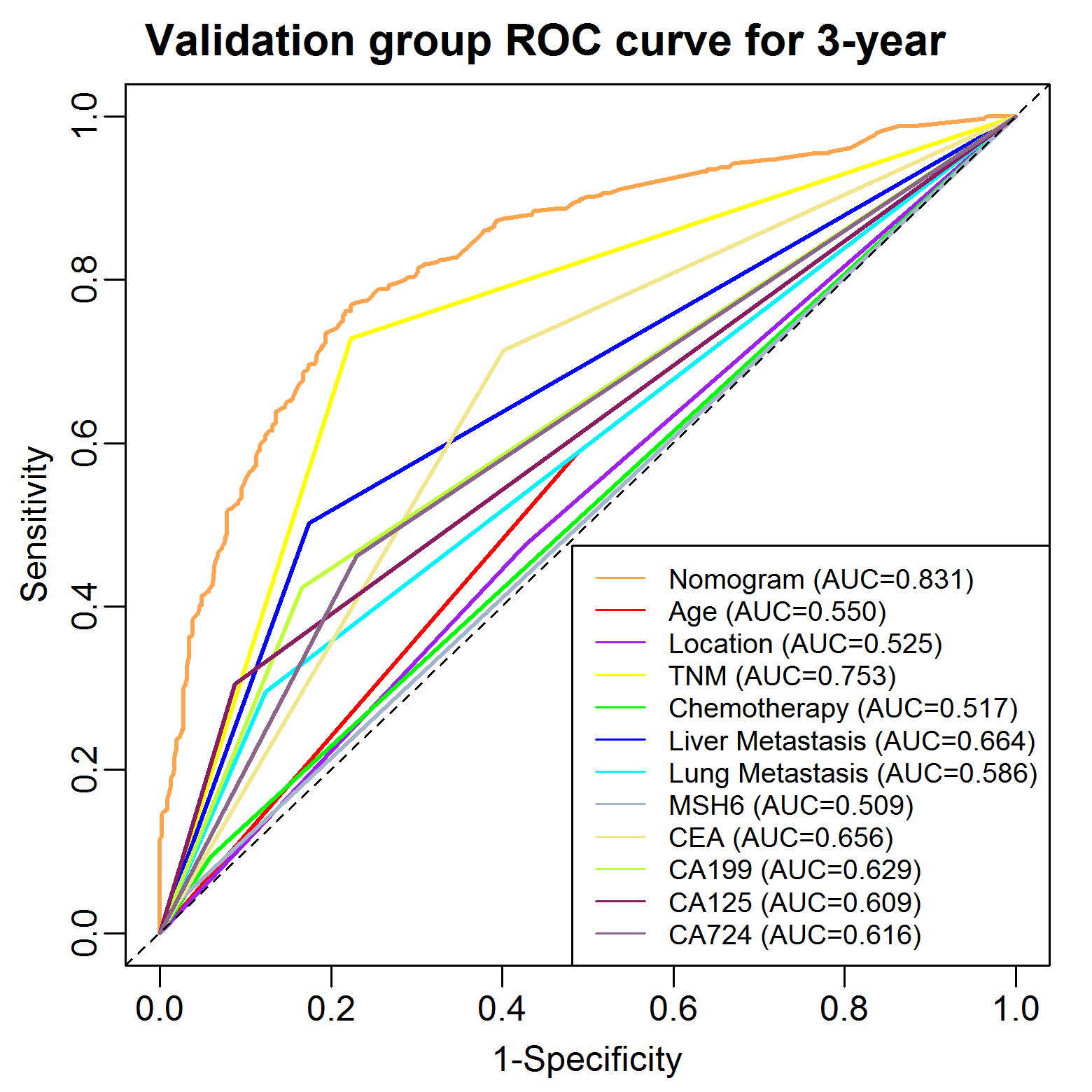


(C) (D)


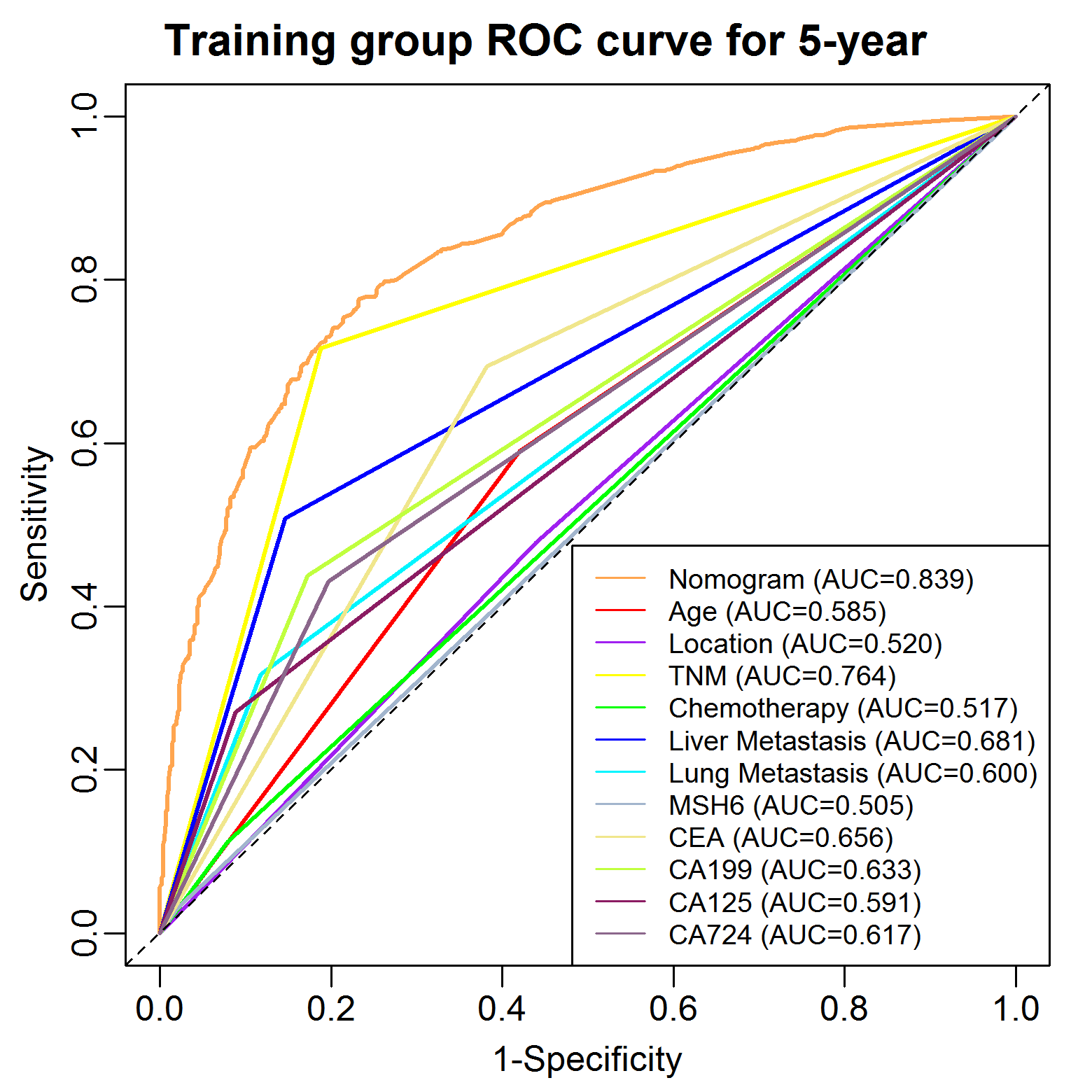

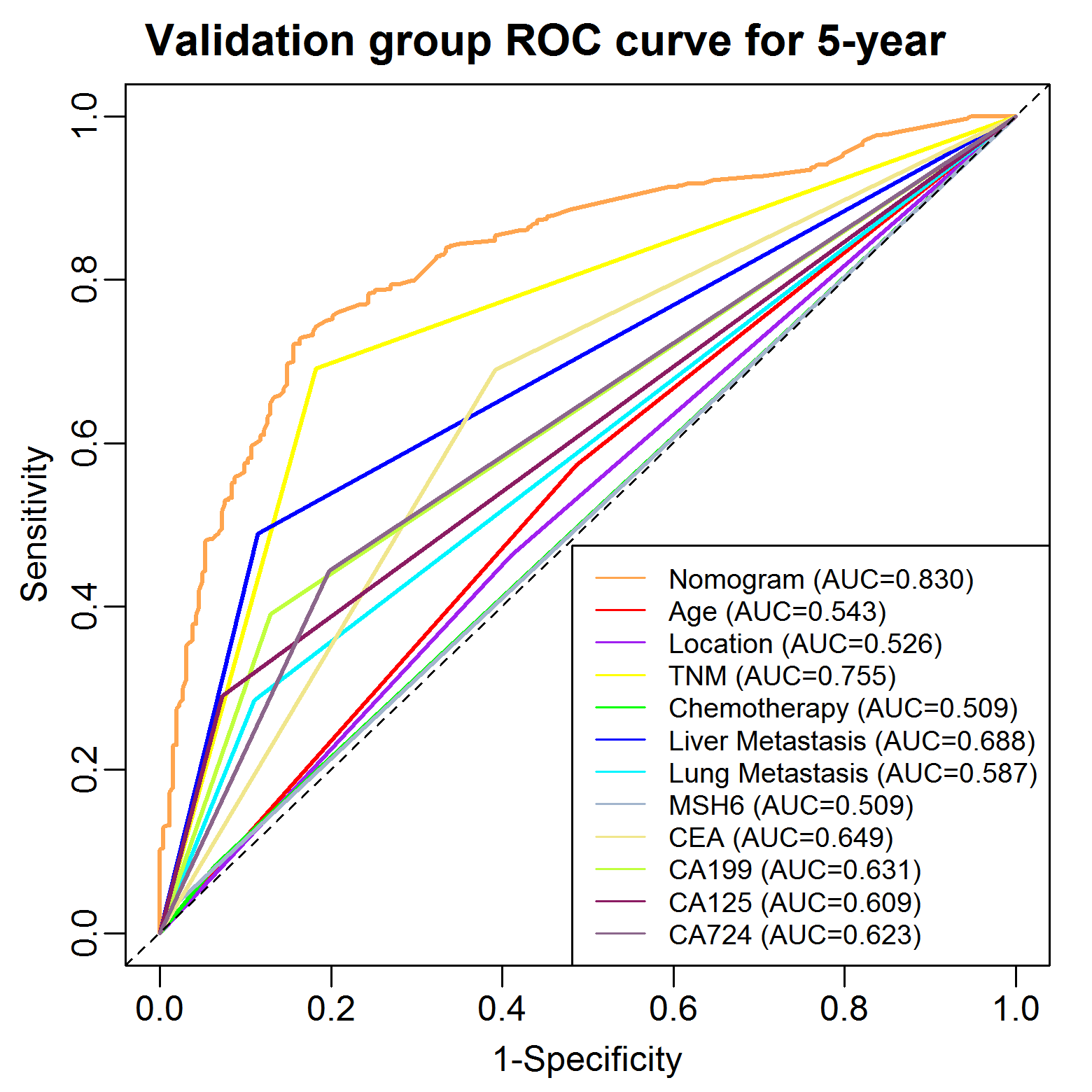


(E) (F)


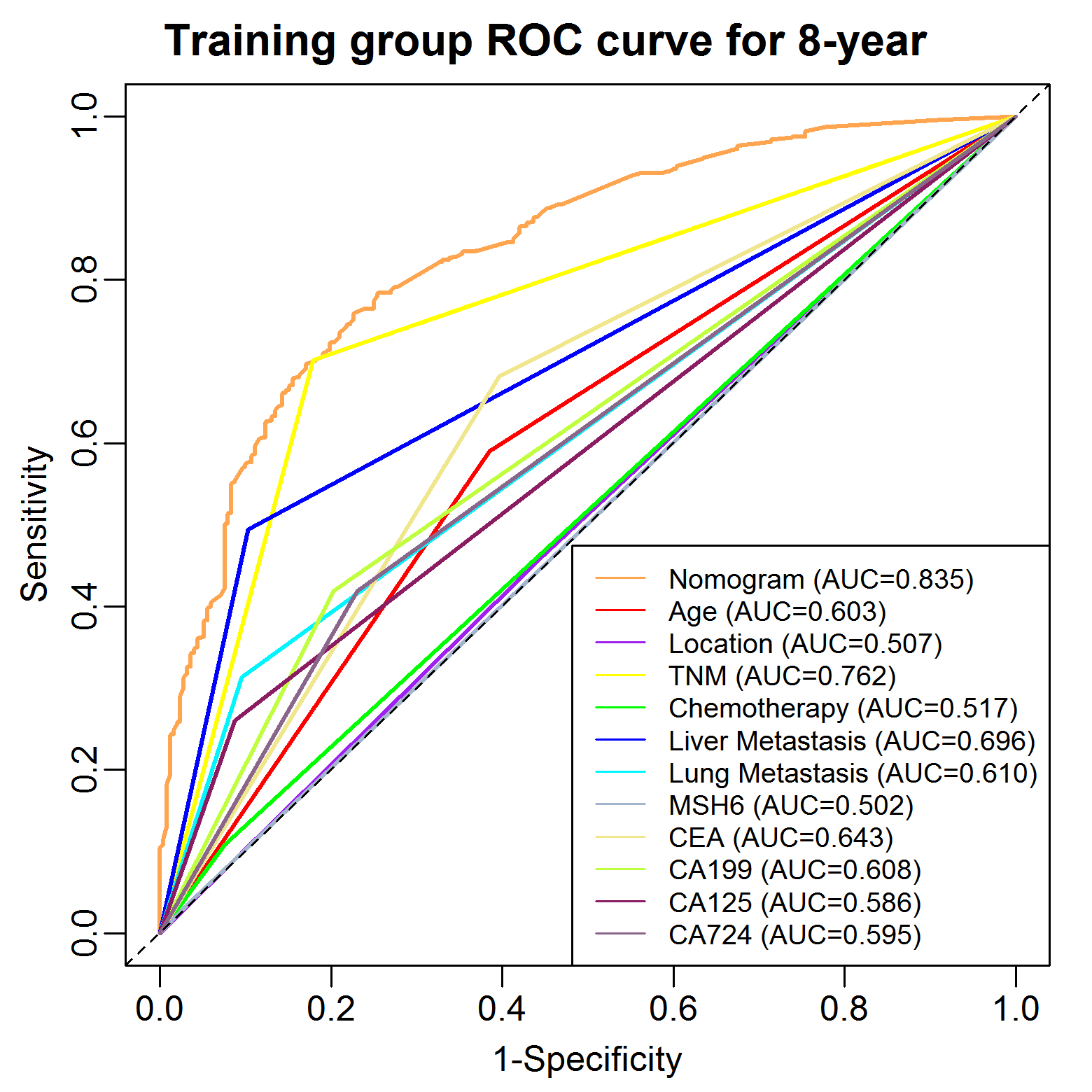

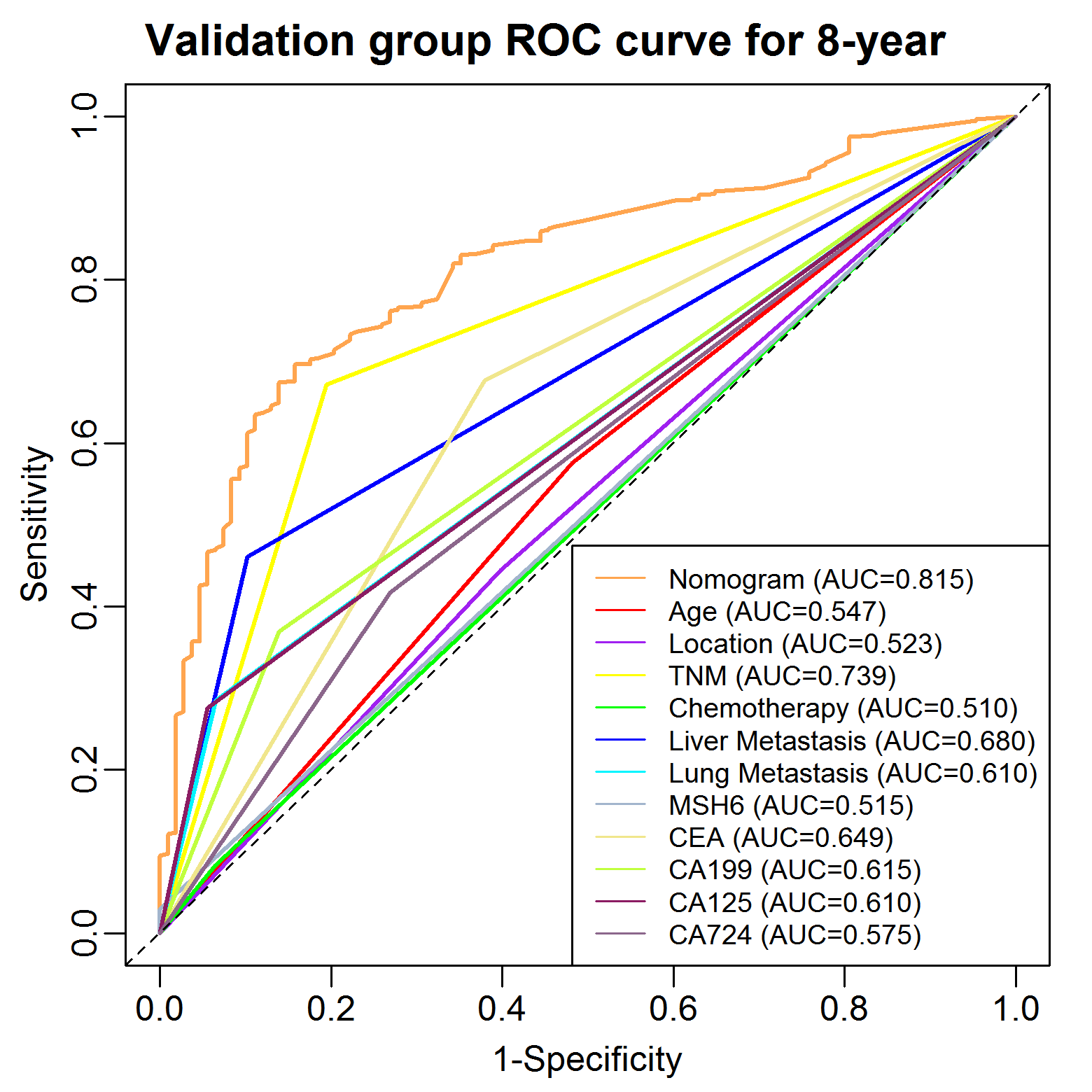


(G) (H)


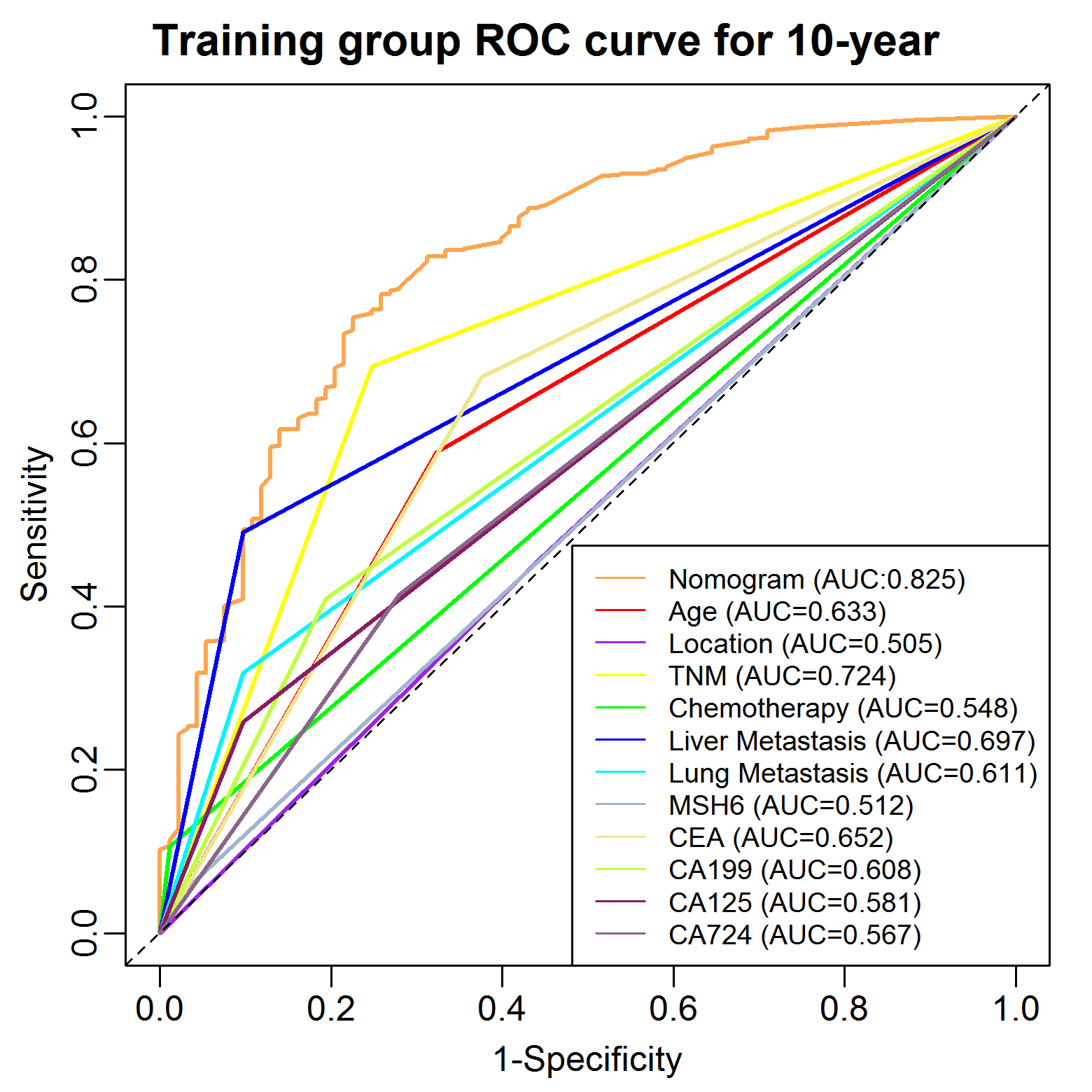

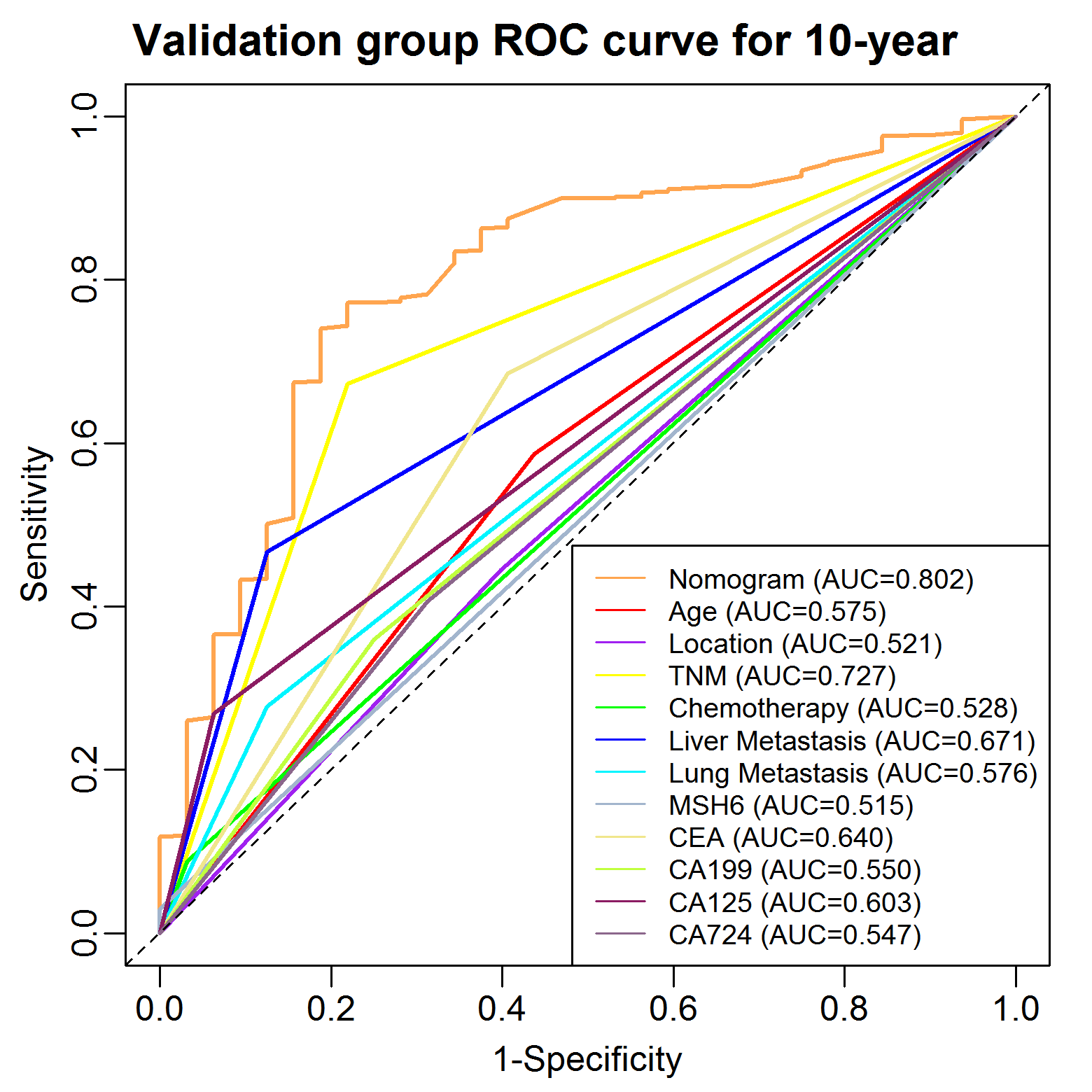


(I) (J)

**FIGURE S1** Nomogram predicting ROC curves for 1-, 3-, 5-, 8-, and 10-year OS in different groups of advanced CRC patients. (A-J) AUC values of the ROC curve for the 11 risk factors in the training and validation groups

(E) (F)
