# Supplementary material for: Establishment and Validation of a Prognostic Nomogram for Predicting Postoperative Overall Survival in Advanced Stage III–IV Colorectal Cancer Patients
Source: Cancer Med. 2024 Nov 15;13(22):e70385. doi: 10.1002/cam4.70385 (PMC11566917; doi:10.1002/cam4.70385)
Supplement: Supplementary file 4 — FIGURE S2. Ten‐year overall survival (OS) Kaplan–Meier curves for advanced stage colorectal cancer (CRC) patients stratified by different risk factors. [file CAM4-13-e70385-s003.docx]

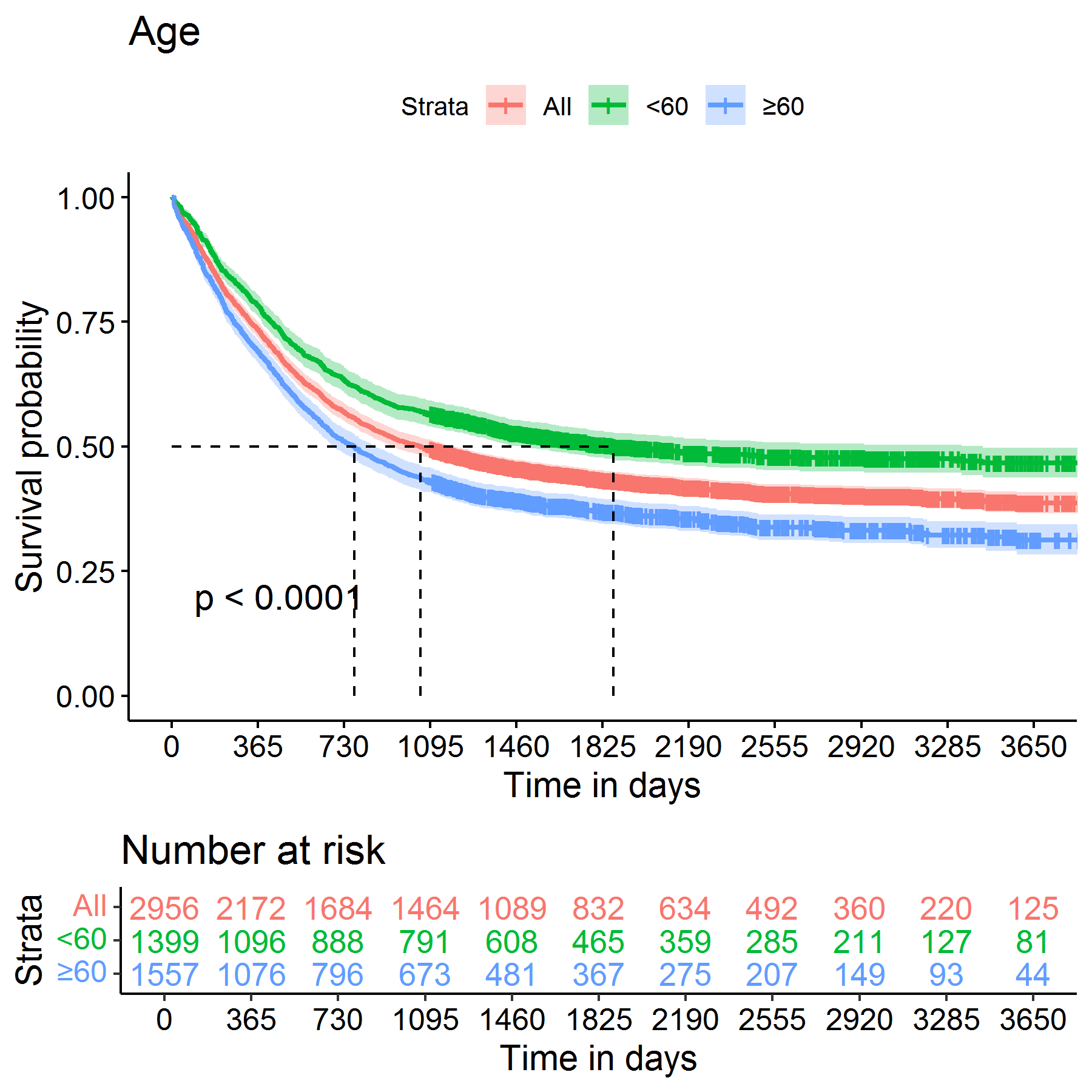

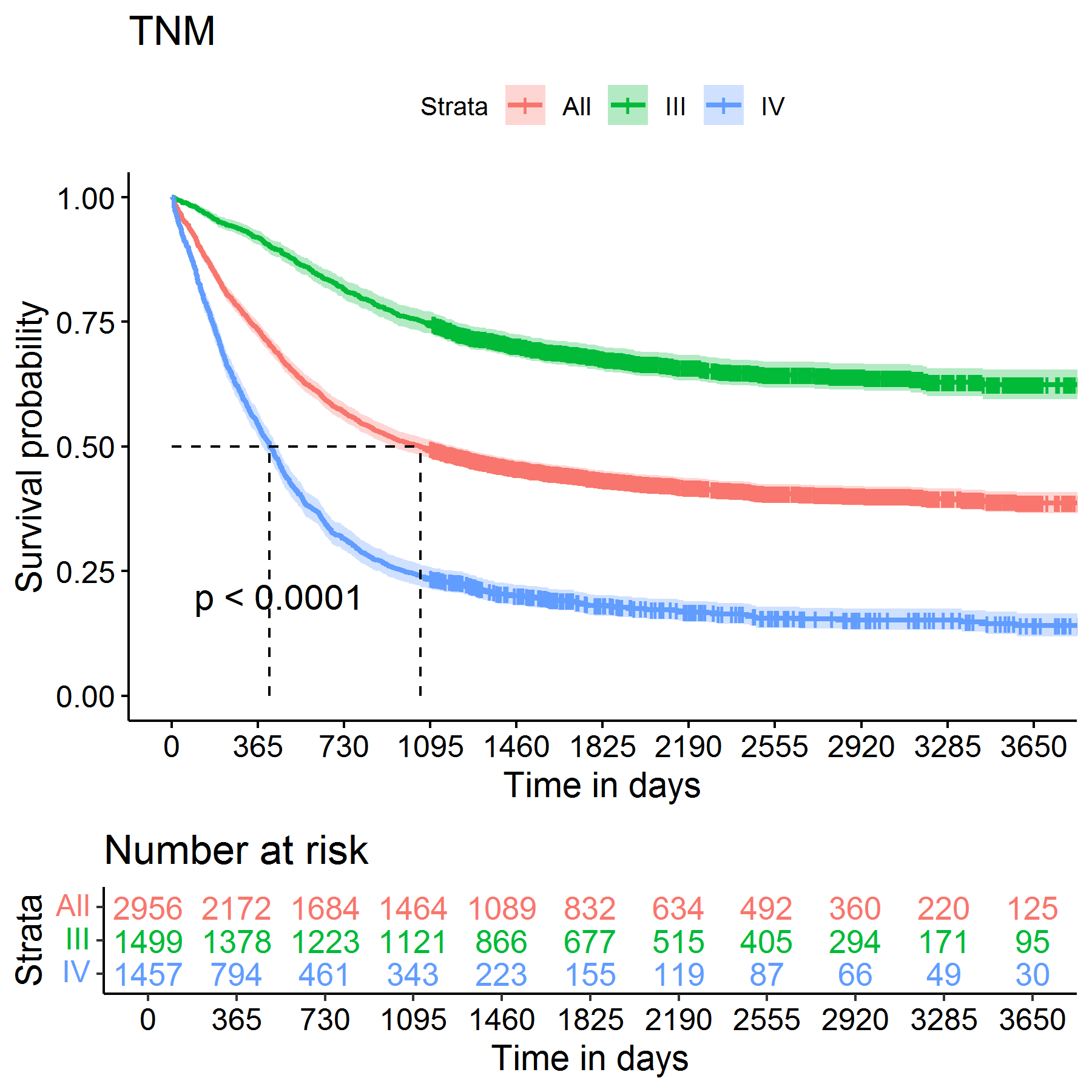


(A) (B)


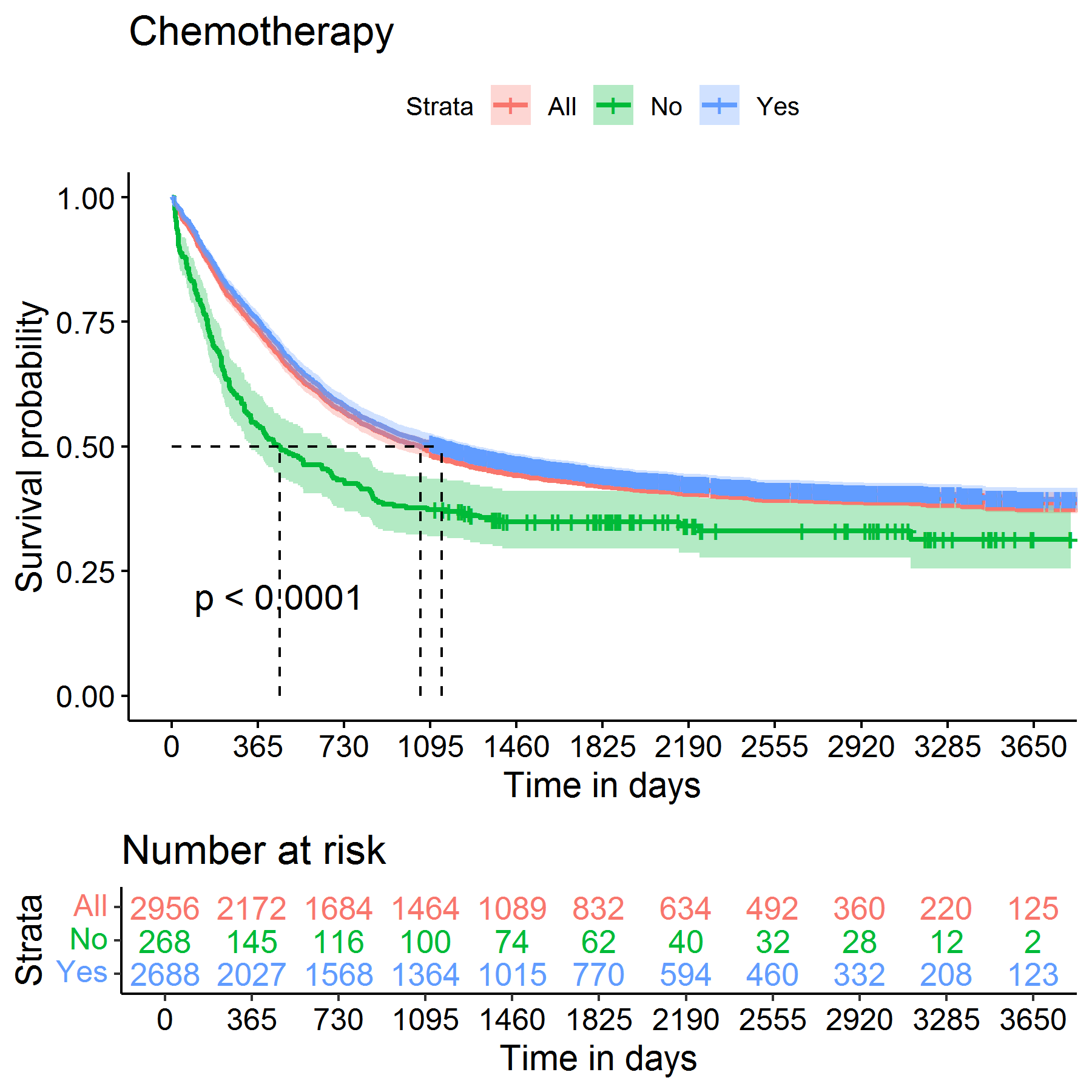

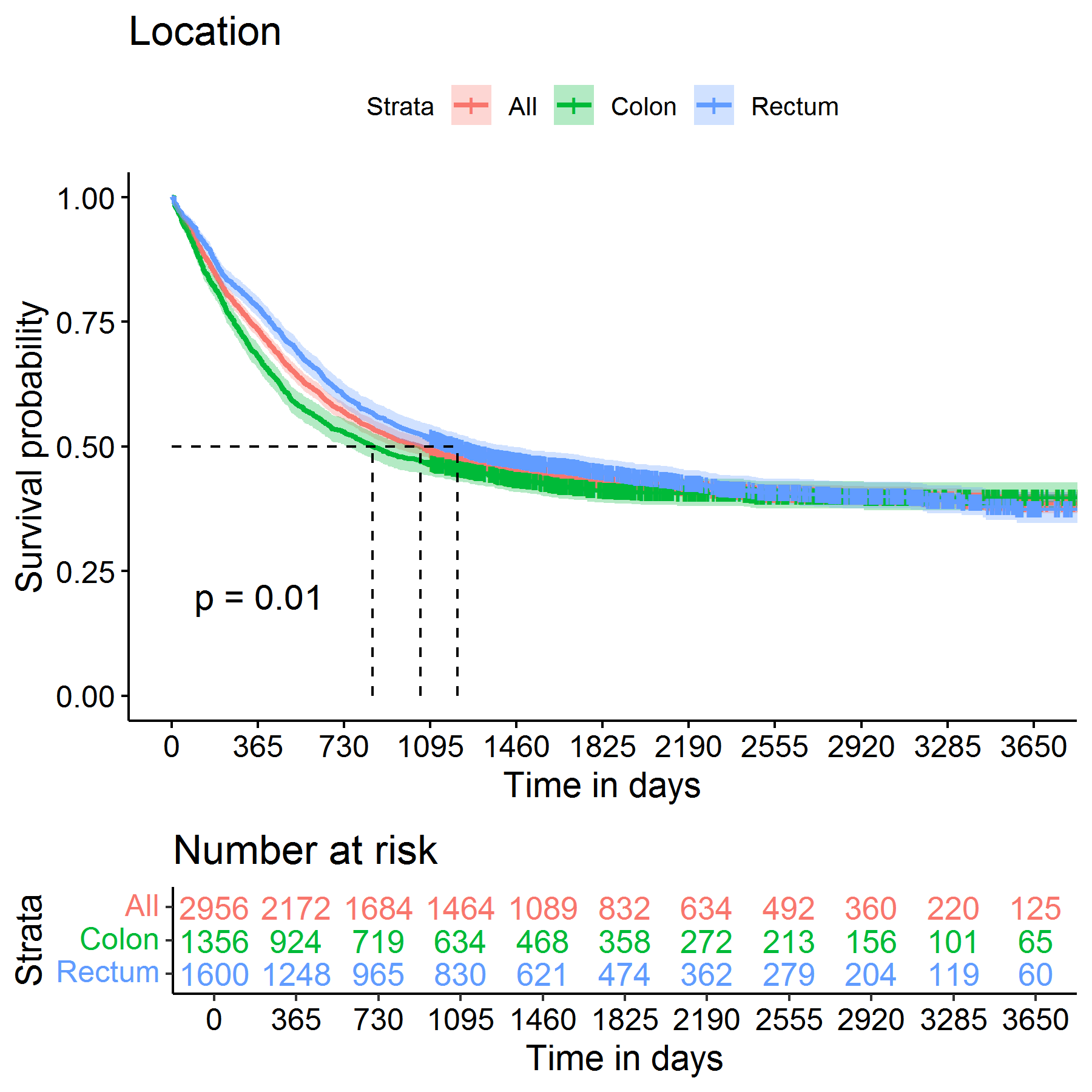


(C) (D)


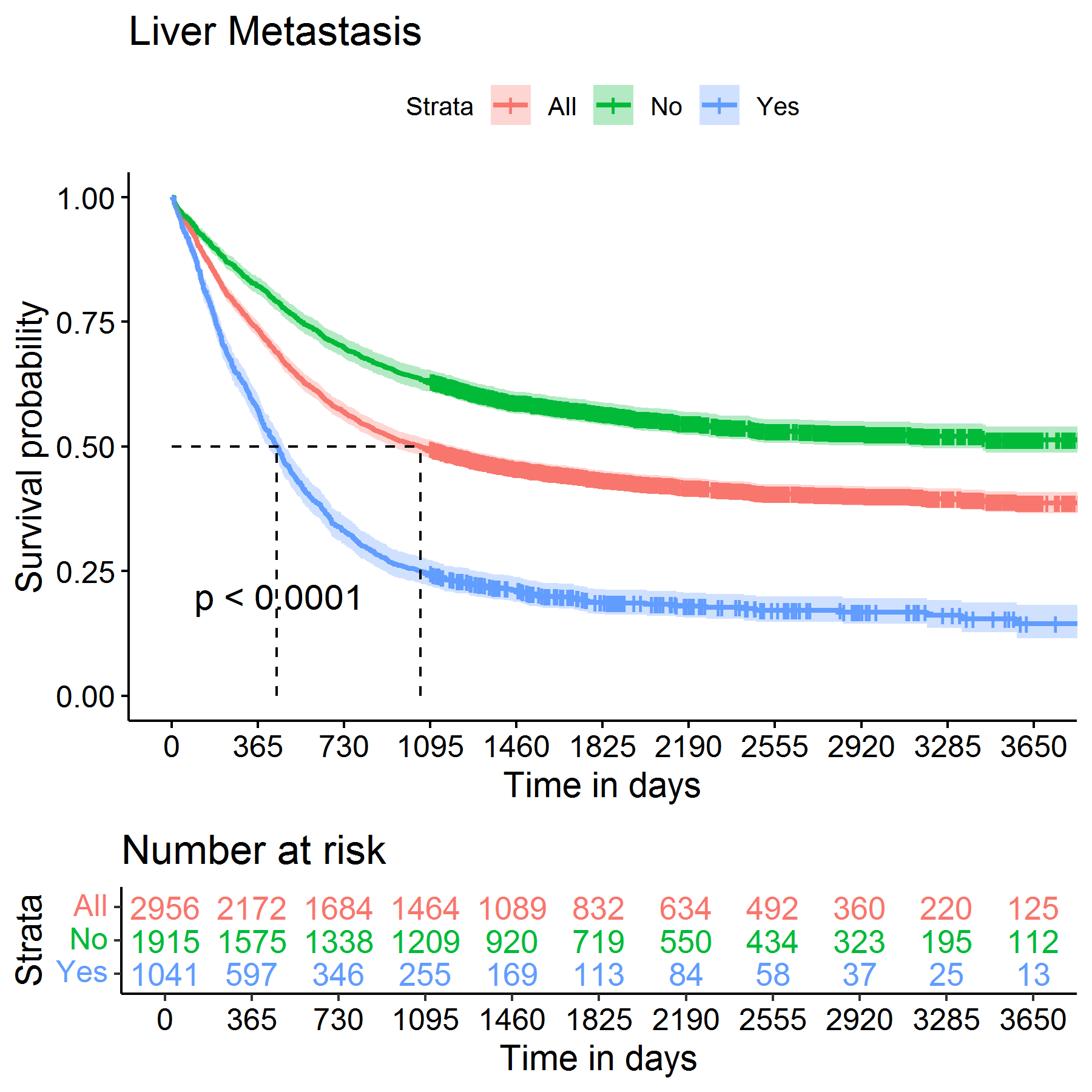

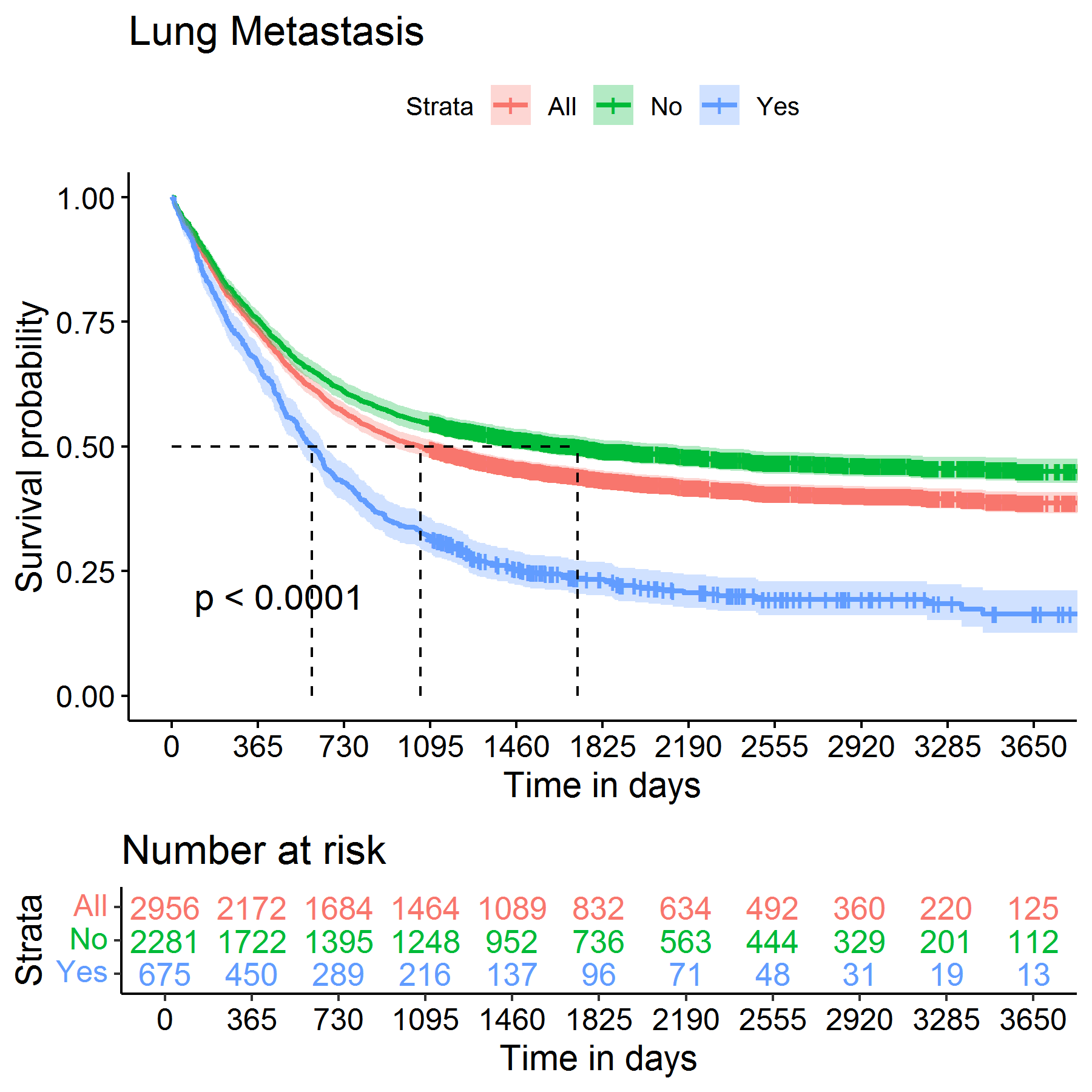


(E) (F)


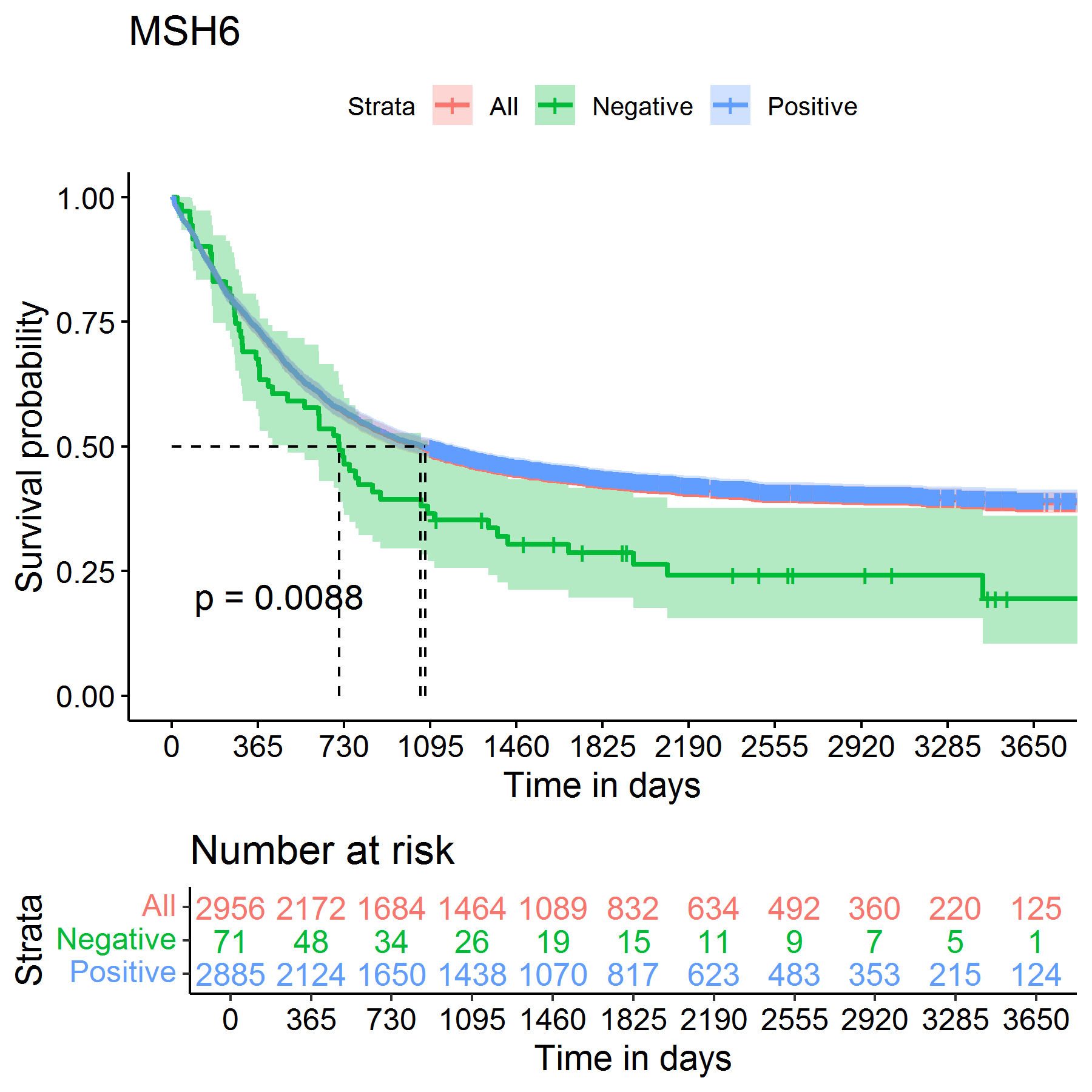

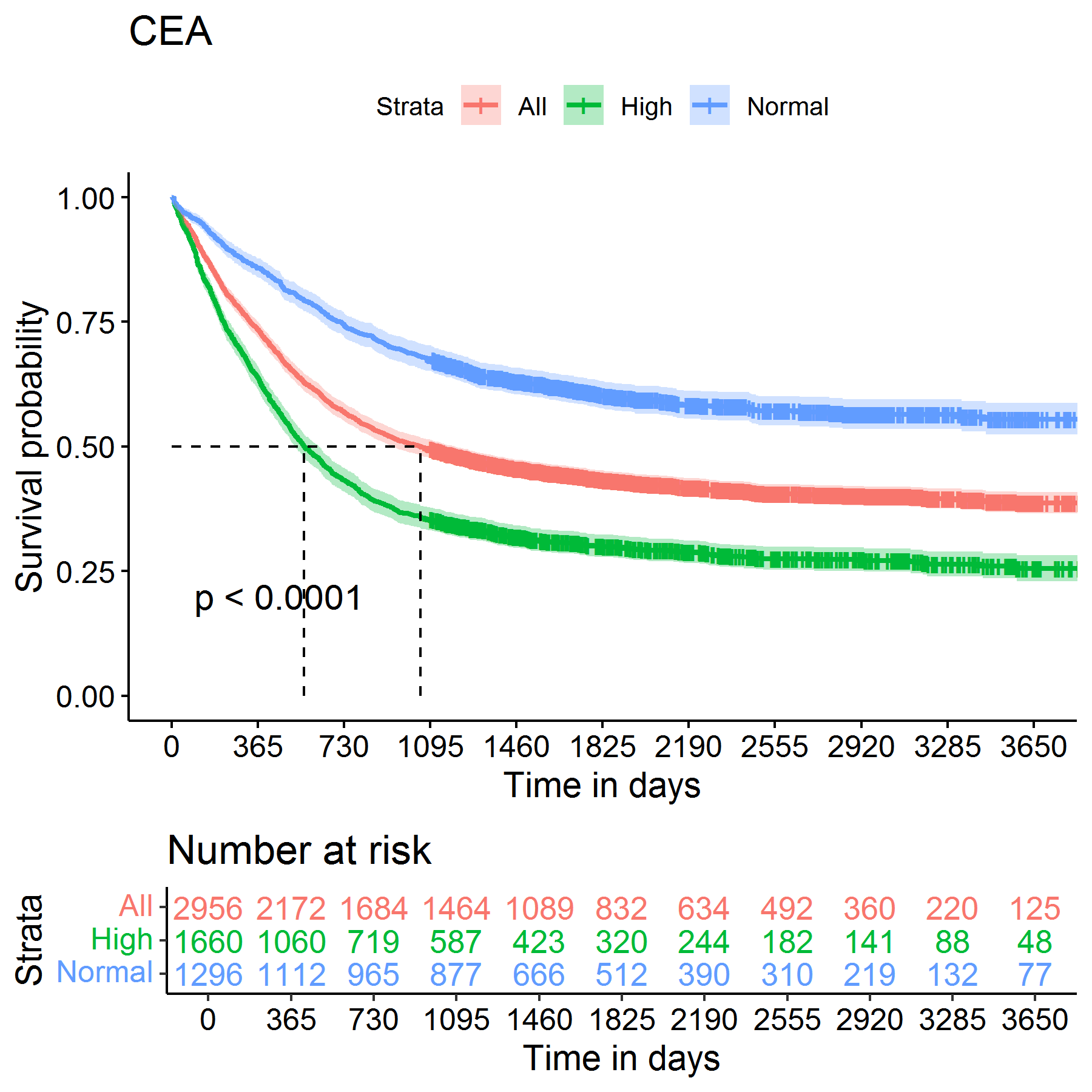


(G) (H)


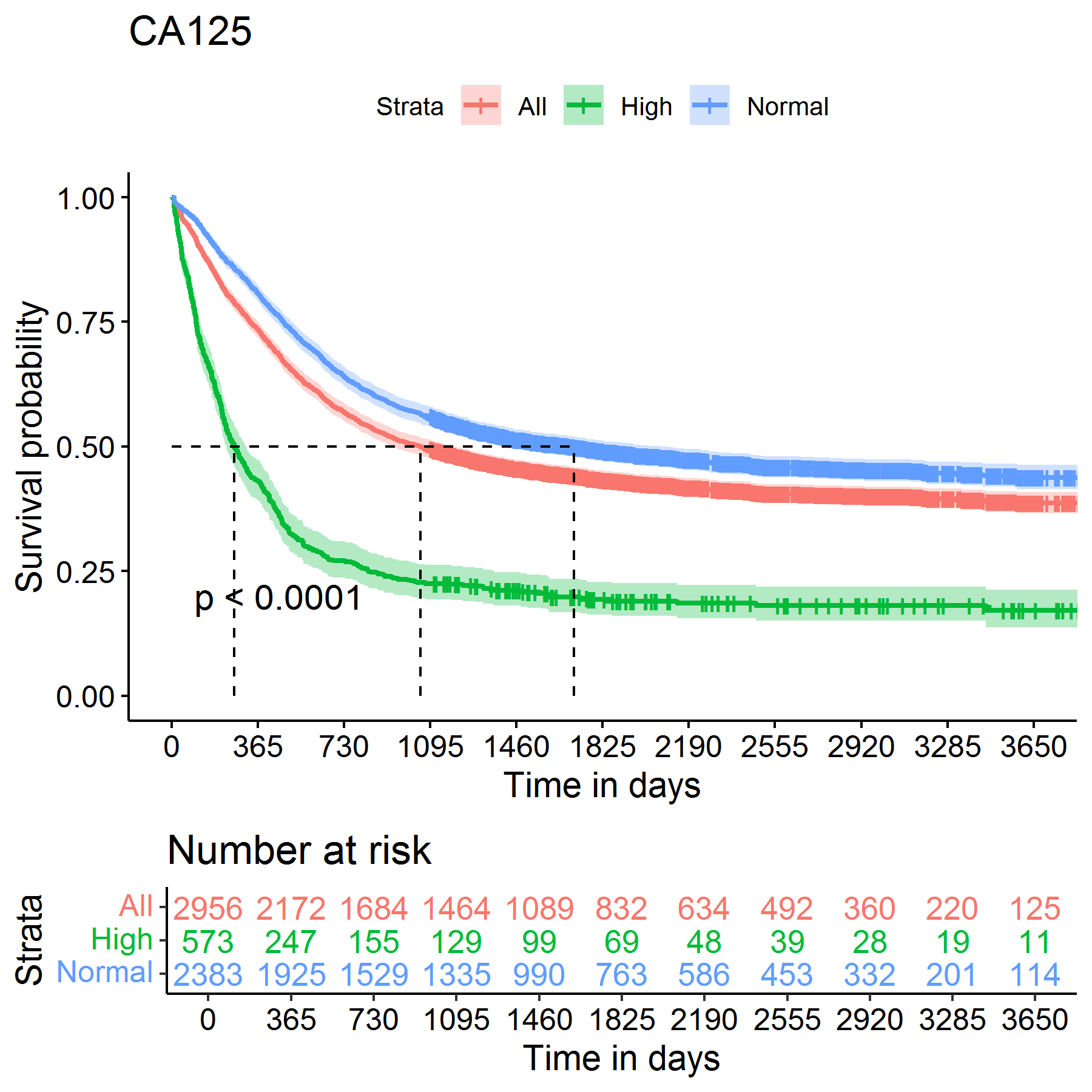

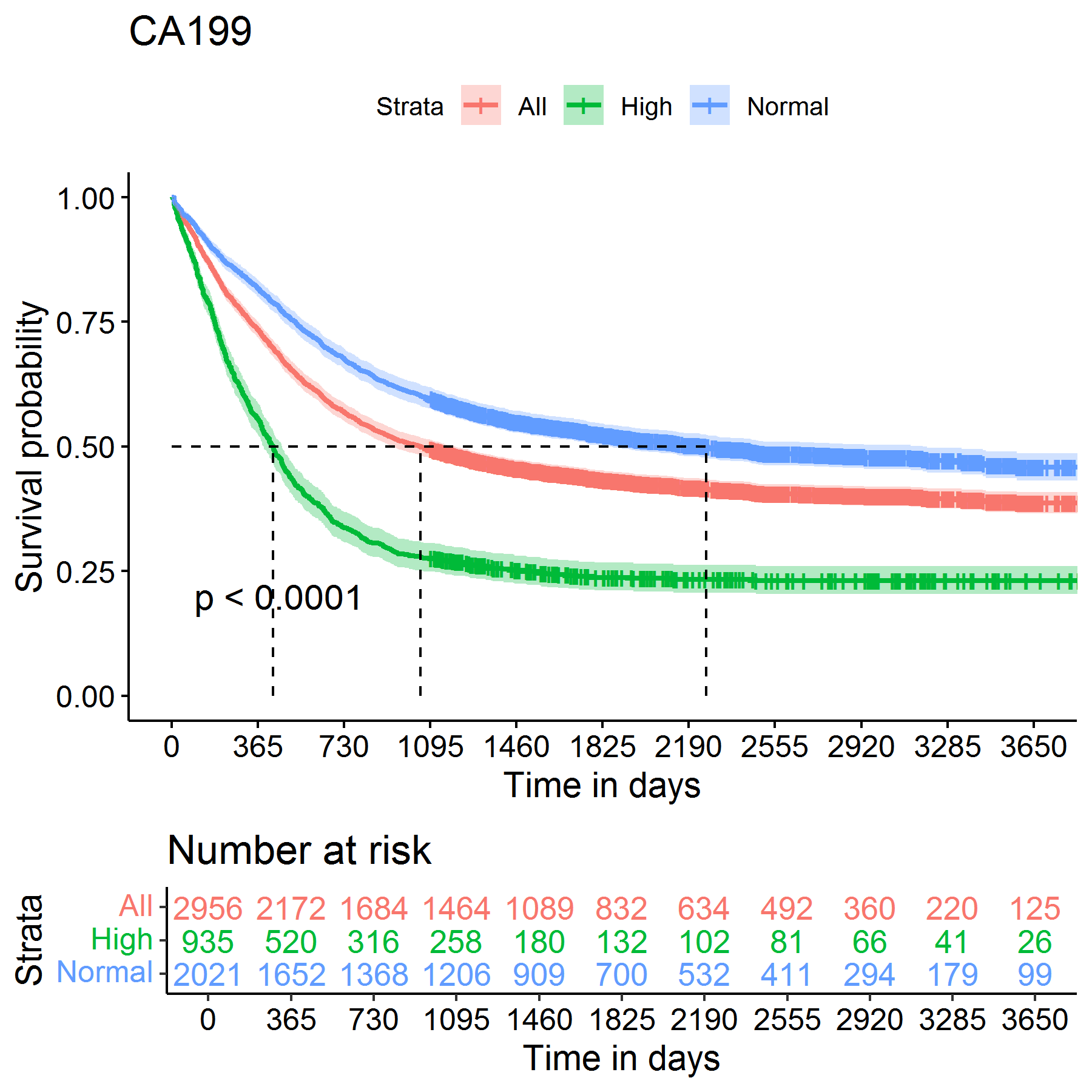


(I) (J)


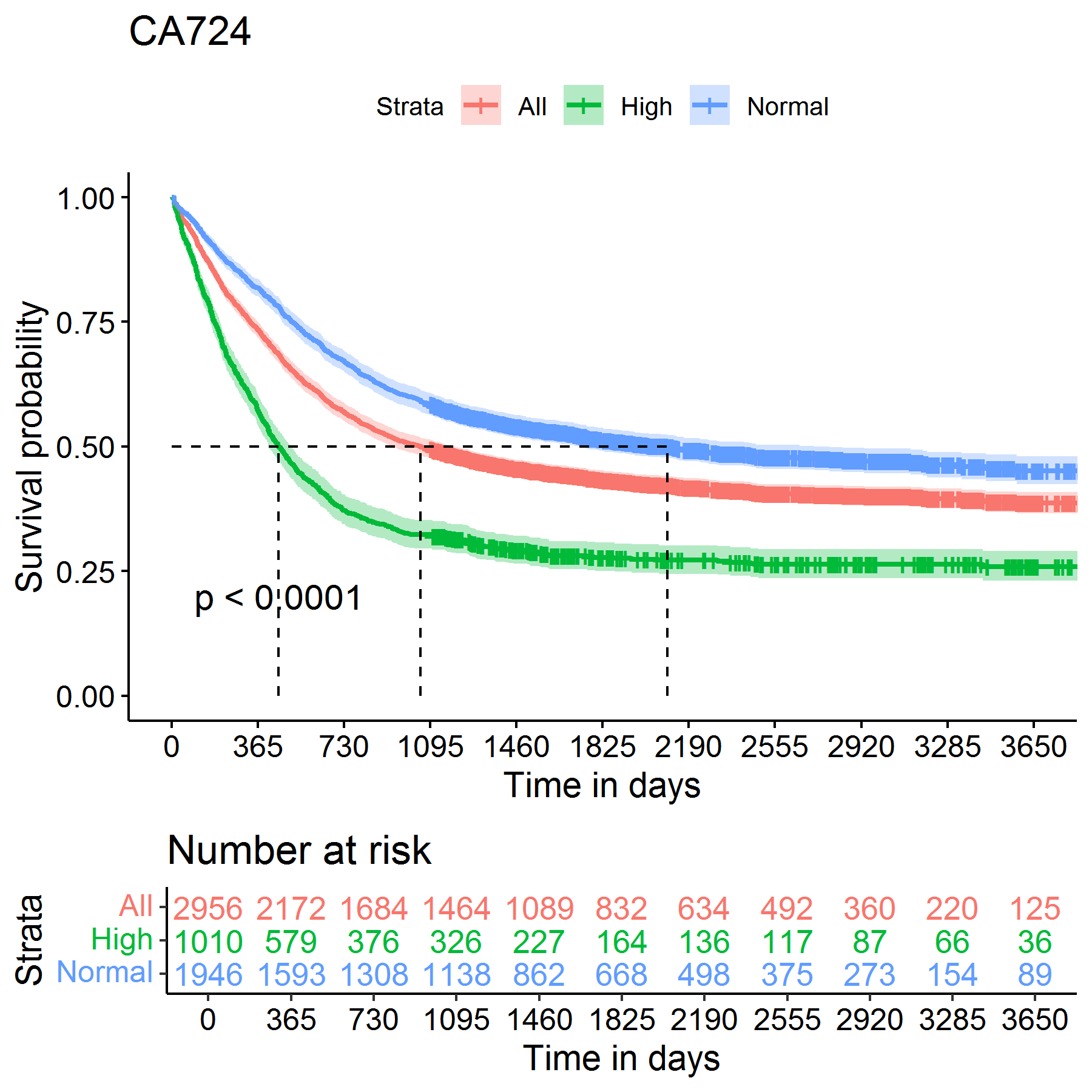


(K)

**FIGURE S2** Ten-year OS Kaplan-Meier curves for advanced CRC patients stratified by different risk factors.
